# scPER: A Rigorous Computational Approach to Determine Cellular Subtypes in Tumors Aligned With Cancer Phenotypes From Total RNA Sequencing

**DOI:** 10.1002/advs.202514502

**Published:** 2025-11-27

**Authors:** Bingrui Li, Xiaobo Zhou, Raghu Kalluri

**Affiliations:** ^1^ Department of Cancer Biology University of Texas MD Anderson Cancer Center Houston TX 77054 USA; ^2^ Center for Computational Systems Medicine School of Biomedical Informatics The University of Texas Health Science Center at Houston Houston TX 77030 USA; ^3^ McGovern Medical School The University of Texas Health Science Center at Houston Houston TX 77030 USA; ^4^ Department of Molecular and Cellular Biology Baylor College of Medicine Houston TX 77030 USA; ^5^ Department of Bioengineering Rice University Houston TX 77005 USA; ^6^ Department of Electrical and Computer Engineering Rice University Houston TX 77005 USA; ^7^ Department of Pathology University of Texas Medical Branch Galveston TX 77555 USA

**Keywords:** cancer biology, deconvolution, machine learning, tumor microenvironment

## Abstract

Single‐cell RNA sequencing (scRNA‐seq) is a powerful technique for understanding cellular diversity, but processing large patient cohorts to identify phenotype‐associated cell populations remains challenging. Here, scPER (**E**stimating cell **P**roportions using **s**ingle‐**c**ell RNA‐seq **R**eference), a rigorous approach combining adversarial autoencoder and extreme gradient boosting to estimate tumor microenvironment cell compositions and identify phenotype‐associated subclusters for bulk RNA‐seq samples. Integrating scRNA‐seq datasets from diverse studies, scPER constructed comprehensive reference panels and disentangled confounders from true signals. scPER achieved superior accuracy in cellular proportion estimation compared to CIBERSORTx, BayesPrism, Scaden, MuSiC, SCDC, DeSide and ReCIDE. It showed high accuracy in predicting metastatic melanoma immunotherapy response and identified a critical T cell subcluster expressing *FCRL3* and *SLAMF7*. In metastatic urothelial cancer, scPER predicted TGFβ‐mediated inhibition of CD4 naïve T cells to diminish PD‐L1 checkpoint blockade efficacy. scPER enables robust integration of scRNA‐seq datasets to estimate cellular proportions across tumors and identify clinically relevant cell populations.

## Graphical abstract

scPER presents an adversarial‐autoencoder framework that deconvolves bulk total RNA‐seq to quantify tumor‐microenvironment cell types and uncover phenotype‐linked subclusters. Across diverse benchmarks, scPER improves accuracy over existing tools. It identifies novel cell subtypes associated with clinical outcomes, and the predicted cell‐type proportions provide a high‐accuracy route to forecasting immunotherapy response. Pretrained panels enable plug‐and‐play use. Created in BioRender. Li, B. (2025) https://BioRender.com/ry4tijs.

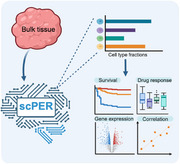

## 1 Introduction

Tumors are complex ecosystems composed of malignant cells and surrounded by diverse cell types, where various interactions take place.^[^ ^1^, ^2^ ^]^ The tumor microenvironment (TME) consists of various immune cells and stromal cells and significantly impacts the growth and progression of the tumor.^[^ ^3^ ^]^ Understanding the composition of the tumor microenvironment is valuable for developing novel cancer therapies and making treatment decisions in clinical practice. In particular, knowledge of the immune cells that infiltrate the tumors can shed light on how the tumor responds to the immune system. It has been demonstrated that spatial location and abundance of immune cells within a patient's tumor are predictive of treatment outcomes for standard therapies.^[^ ^4^, ^5^, ^6^ ^]^ Similarly, the presence of certain T cell subsets at the site of the tumor has been shown to be correlated with the effectiveness of checkpoint inhibitor immunotherapies such as anti‐PD1, anti‐PDL1, and anti‐CTLA4.^[^ ^7^ ^]^ Therefore, it is thought that the patient‐specific immune cell composition within a tumor may be a key factor in predicting response to immunotherapy. This information can be used to stratify the patients in clinical trials and predict treatment options to target specific immune cell types, improving the chances of success and accelerating access to more effective treatment options.^[^ ^1^ ^]^ Fluorescence‐activated cell sorting (FACS) and immunohistochemistry are both highly regarded techniques for quantifying the immune cell content within a sample.^[^ ^8^ ^]^ However, they both generally rely on a small number of combinations of preselected marker genes, which limits the number of cell types that can be quantified simultaneously. More recently, single‐cell RNA sequencing (scRNA‐seq) technologies have revolutionized biomedical research by profiling the transcriptomic landscape of thousands of individual cells and enabled researchers to describe cell types and states. However, its implementation in clinical settings remains cost‐prohibitive and labor‐intensive, rendering routine use infeasible. Moreover, most clinical specimens cannot be dissociated into single cells for scRNA‐seq analysis, and variations in the efficiency of single‐cell dissociation can lead to bias in the quantification of cell type proportions in the scRNA‐seq data.^[^ ^9^ ^]^ Moreover, the existing scRNA‐seq technology lacks the capability to associate cell populations with clinical phenotypes of tumors, which are primarily obtained from bulk tissue samples, particularly in the form of formalin‐fixed, paraffin‐embedded samples that are incompatible with single‐cell analysis.

Recently, various computational approaches have been described to estimate the cellular subsets in tumors.^[^ ^10^, ^11^, ^12^, ^13^, ^14^, ^15^, ^16^, ^17^, ^18^, ^19^ ^]^ The majority of these methods use either cell type‐specific marker genes derived from the FACS‐purified cell subsets, or the gene expression profiles (GEPs) inferred by the deconvolution methods to estimate the cell proportions in bulk samples.^[^ ^10^, ^11^, ^20^, ^21^, ^22^ ^]^ Recent research has demonstrated that the design of a GEP is a crucial aspect in most methods used to unravel mixed cell populations, as comparable results can be obtained using different methods with the same GEP.^[^ ^23^ ^]^ Ideally, the signature genes included in the optimal GEP should be highly specific to individual cell populations in a complex sample.^[^ ^24^ ^]^ Furthermore, they should maintain stability across varying conditions, such as in health and disease states, and exhibit robustness against noise or biases in the data. However, due to the inherent biases presented in biomedical data, such as variations across tissues, individuals, species, and data acquisition methods, it is typically challenging to achieve optimal performance in cell‐type deconvolution.^[^ ^18^ ^]^ In addition, the GEP‐based deconvolution approach encountered difficulty in differentiating the cell subtypes and estimating their proportions well.^[^ ^25^ ^]^ Alternatively, various deep learning‐based approaches have been described for estimating cell proportions by automatically extracting features from simulated scRNA‐seq data instead of relying on a pre‐defined GEP.^[^ ^18^, ^26^ ^]^ However, these strategies generally concentrate on individual scRNA‐seq data to uncover features specific to the cell types reported. Nonetheless, the single dataset often lacks representation of all required cell types and the scRNA‐seq data may not be accessible for certain tissues, necessitating the deconvolution approach to possess the capability to integrate multiple scRNA‐seq datasets and perform inter‐tissue prediction. However, references assembled from multiple scRNA‐seq studies can carry batch and platform effects that degrade accuracy on external bulks. Methods such as MuSiC^[^ ^27^ ^]^ and SCDC^[^ ^28^ ^]^ typically assume that all target cell types are present in each reference dataset, which is often not satisfied in TME atlases, and within‐tissue tuning can limit cross‐tissue generalization when a matched single‐cell atlas is unavailable. Moreover, most frameworks are not designed to connect deconvolved proportions to clinical endpoints or to discover phenotype‐linked subclusters in a single pipeline, and deep learning methods may overfit without explicit confounder control.

To address the aforementioned challenges, we proposed a novel approach for **E**stimating the cell **P**roportions using the **s**ingle‐**c**ell RNA‐seq **R**eference (scPER) and identifying the phenotype‐associated subclusters. This approach comprises two components: an adversarial deconfounding autoencoder model, which disentangles the confounding factors from the true signals and generates the biologically informative embeddings of scRNA‐seq datasets derived from different studies and tissues and an eXtreme Gradient Boosting (XGBoost) regression model, which estimates the proportions of the embedded cell types in the bulk samples. Upon the cell fractions estimated by scPER, we further identified the biologically informative cell subpopulations associated with the clinical phenotypes, such as immunotherapy response. We also demonstrated that the cell fractions estimated by scPER can be used to predict the immunotherapy response of patients with high accuracy, enabling the possibility of stratifying patients in clinical settings.

## 2 Results

### 2.1 Overview of the scPER Framework

**Figure 1:**
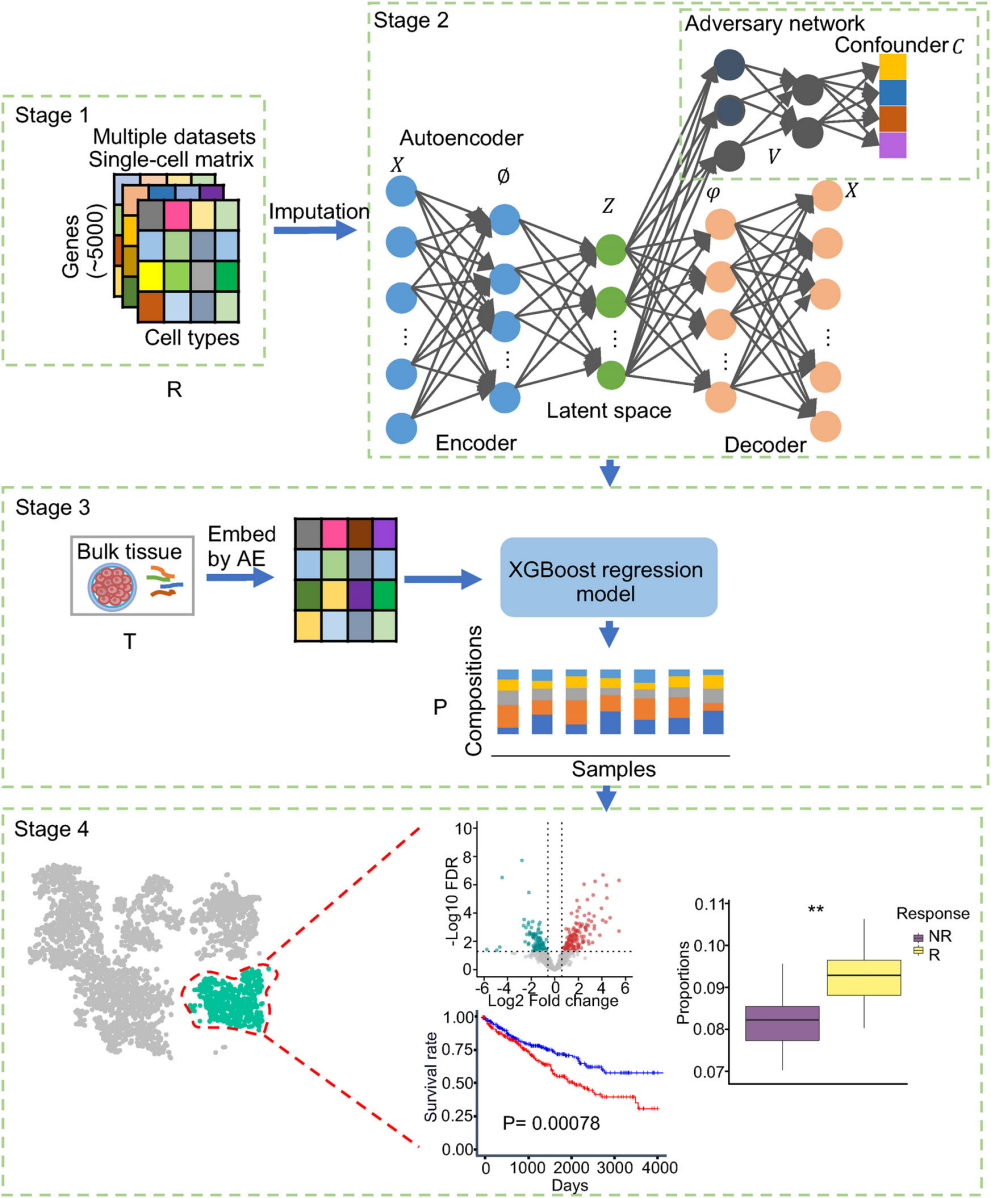
Overview of scPER and the function of each stage. Stage 1. Reference integration and preprocessing. scRNA‐seq datasets from multiple studies or tissues are integrated to form a reference matrix *R* (rows = genes, columns = cells). After QC and feature selection, denoising and imputation are applied to stabilize gene signals across datasets. Output: preprocessed single‐cell expression *R* with cell‐type labels and confounder labels (study/platform/tissue). Stage 2. Confounder‐aware representation learning. An adversarial autoencoder learns a low‐dimensional latent space *Z* from *R*. The encoder *E* maps expression *x* to *z*; the decoder *D* reconstructs *x* from *z*. An adversary *V* tries to predict designated confounders *c* (e.g., study/platform/tissue) from *z*. Training minimizes reconstruction while implicitly maximizing adversary loss with weight λ (i.e., *L*_rec_ − λ*L*_adv_), yielding *Z* that preserves biology but reduces nuisance variation. Output: cell embeddings *Z* and a trained encoder *E* for embedding new data. Stage 3. Bulk embedding and proportion estimation. Bulk RNA‐seq samples *T* are normalized like the single‐cell data and passed through the trained encoder *E* to obtain bulk embeddings in the same latent space. An XGBoost regressor is trained on latent features from single‐cell references to predict cell‐type compositions *P* for each bulk sample (columns = samples, rows = cell types). Output: estimated cell‐type proportions *P* across all bulk samples. Stage 4. Downstream biology and clinical association. The estimated compositions *P* enable downstream analyses: (i) identify critical genes modulating the cell proportions, (ii) test clinical endpoints such as survival or response to immunotherapy, and (iii) identify phenotype‐associated changes in cell types or subclusters. *R* = reference single‐cell matrix; *T* = bulk expression; *Z* = scPER latent embeddings; *P* = estimated cell‐type proportions; *λ *= adversarial weight; *c* = confounder label. Arrows indicate data flow from reference construction to embedding, deconvolution, and phenotype analyses.

scPER was developed to characterize the fractions of cell types in the bulk samples using batch‐corrected latent representations embedded by large‐scale, diverse single‐cell transcriptomic profiles (**Figure** **1**). To achieve this goal, our strategy combines the adversarial autoencoder and XGBoost to generate the biologically informative embeddings of single‐cell transcriptomic profiles while minimizing out‐of‐interest sources of variations, such as technical factors and tissue and cancer‐type variations. The adversarial autoencoder comprises two neural networks training simultaneously (Figure 1). The autoencoder model aims to generate an embedding that accurately reconstructs the transcriptomic profiles of the various scRNA‐seq datasets. Meanwhile, the adversary network is trained to identify the confounders from the embeddings generated by the autoencoder. These two networks are trained against each other to determine the optimal embeddings that incorporate critical signals from the single‐cell transcriptomic profiles while disregarding the variations of the chosen confounders. Upon the optimal embeddings of both scRNA‐seq and bulk samples, we then adopted the XGBoost regression model to estimate the proportions of those cell types in bulk RNA‐seq mixtures. The cell fractions estimated by scPER were applied to identify the phenotype‐associated cell populations and understand the mechanisms of disease progression and displayed a high accuracy in predicting the immunotherapy response of patients.

### 2.2 Robust Deconvolution of Bulk Peripheral Blood Mononuclear Cells with Matched scRNA‐seq Data

**Figure 2:**
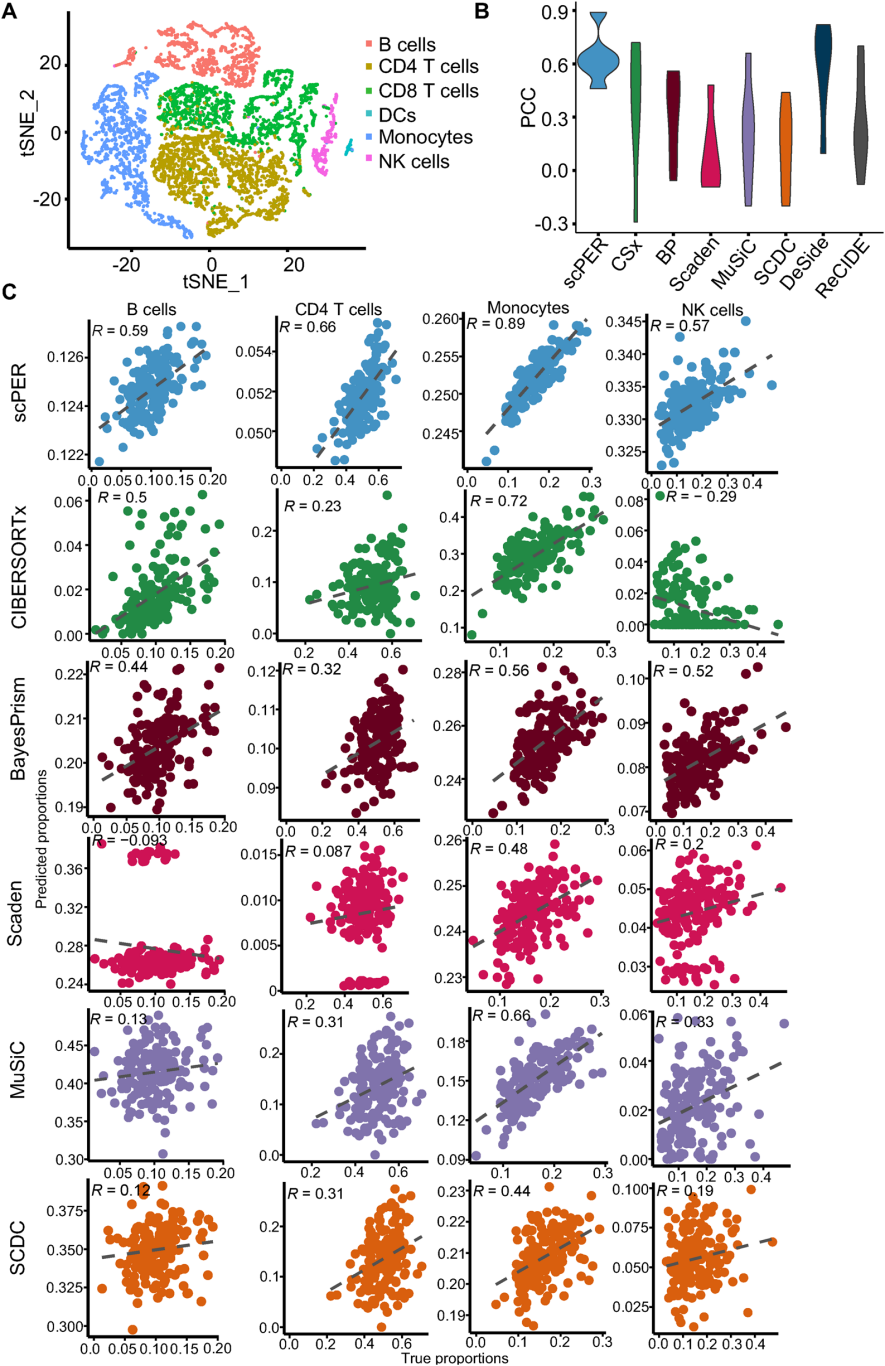
Benchmarking deconvolution accuracy on PBMC single‐cell and bulk profiles.(A) t‐Distributed Stochastic Neighbor Embedding (t‐SNE) of the 100 latent embeddings learned from scRNA‐seq data, with points colored by the six annotated immune cell types. (B) Pearson correlation coefficients (*r*) between true and predicted cell‐type fractions across all bulk samples, shown for each deconvolution tool as a violin plot summarizing the distribution of *r* values. (C) True versus predicted proportion scatter plots for each cell type and method. Each point represents one bulk sample (n = 164); the x‐axis shows the ground‐truth fraction (flow cytometry), and the y‐axis shows the fraction predicted by the deconvolution tool.

We began by constructing the scRNA‑seq reference panel from peripheral blood mononuclear cells (PBMCs) obtained from a healthy donor in the 10× Genomics dataset.^[^ ^29^ ^]^ Unsupervised clustering and cell type annotation identified six major leukocyte cell types (B cells, CD4 T cells, CD8 T cells, DCs, Monocytes and NK cells; Figure S1A, Supporting Information). To evaluate the performance of scPER, we selected the top 5,000 most variable genes from the scRNA‐seq data and embedded them into 100 latent dimensions to differentiate among cell types. The matrix of 100 latent dimensions represents the major components of the transcriptomic profile and can effectively differentiate the six cell types (**Figures 2**A; S1B, Supporting Information). We then encoded a validation cohort comprising bulk whole blood microarray profiles from 164 adults sampled before and after vaccination, with corresponding cell proportions measured by flow cytometry (Table S1, Supporting Information),^[^ ^30^ ^]^ and used scPER to predict the proportions of the six cell types in these samples. We compared scPER with seven state‐of‐the‐art cell deconvolution approaches, including CIBERSORTx^17^ (CSx), BayesPrism^[^ ^31^ ^]^ (BP), single cell‐assisted deconvolutional DNN^[^ ^18^ ^]^ (Scaden), Multi‐subject Single Cell deconvolution^[^ ^27^ ^]^ (MuSiC), SCDC,^[^ ^28^ ^]^ DeSide^[^ ^21^ ^]^ and ReCIDE.^[^ ^22^ ^]^ We evaluated their performance by comparing the estimated proportions with the ground truth as determined by flow cytometry in the original study.^[^ ^30^ ^]^ scPER demonstrated the highest overall Pearson correlation (PCC) and the lowest mean squared error (MSE) and mean absolute deviation (mAD) compared with CIBERSORTx, BayesPrism, Scaden, MuSiC, SCDC, DeSide, and ReCLIDE (Figures 2B; S1C,D and Table S2, Supporting Information). These metrics indicate that scPER estimates cell‐type proportions more accurately than existing methods. In particular, its lower mAD values reflect more accurate cell‐proportion predictions across bulk samples. Moreover, upon closer examination of each cell type, scPER consistently achieved the highest PCC and did not exhibit any negative correlations compared to other approaches (Figures 2C; S2A,B, Supporting Information).

### 2.3 scPER Enables Accurate Cross‐Study Estimation of Tumor Microenvironment Components in Pancreatic Cancer

To comprehensively profile bulk samples, scPER needs to correct for inter‑study batch effects and generalize across multiple scRNA‑seq datasets to include all relevant cell types. With this in mind, we applied scPER to characterize the pancreatic tumor microenvironment by predicting the proportions of key cell populations, including B cells, CD8 T cells, endothelial cells, fibroblasts, and monocytes/macrophages, in pancreatic cancer samples. Accurate deconvolution requires a reference panel that contains all relevant cell types, but individual scRNA‑seq studies typically sample only a subset of these populations. To address this challenge, we leveraged scPER to integrate multiple pancreatic cancer scRNA‐seq datasets, thereby constructing a comprehensive reference that captures all relevant cell types.

**Figure 3:**
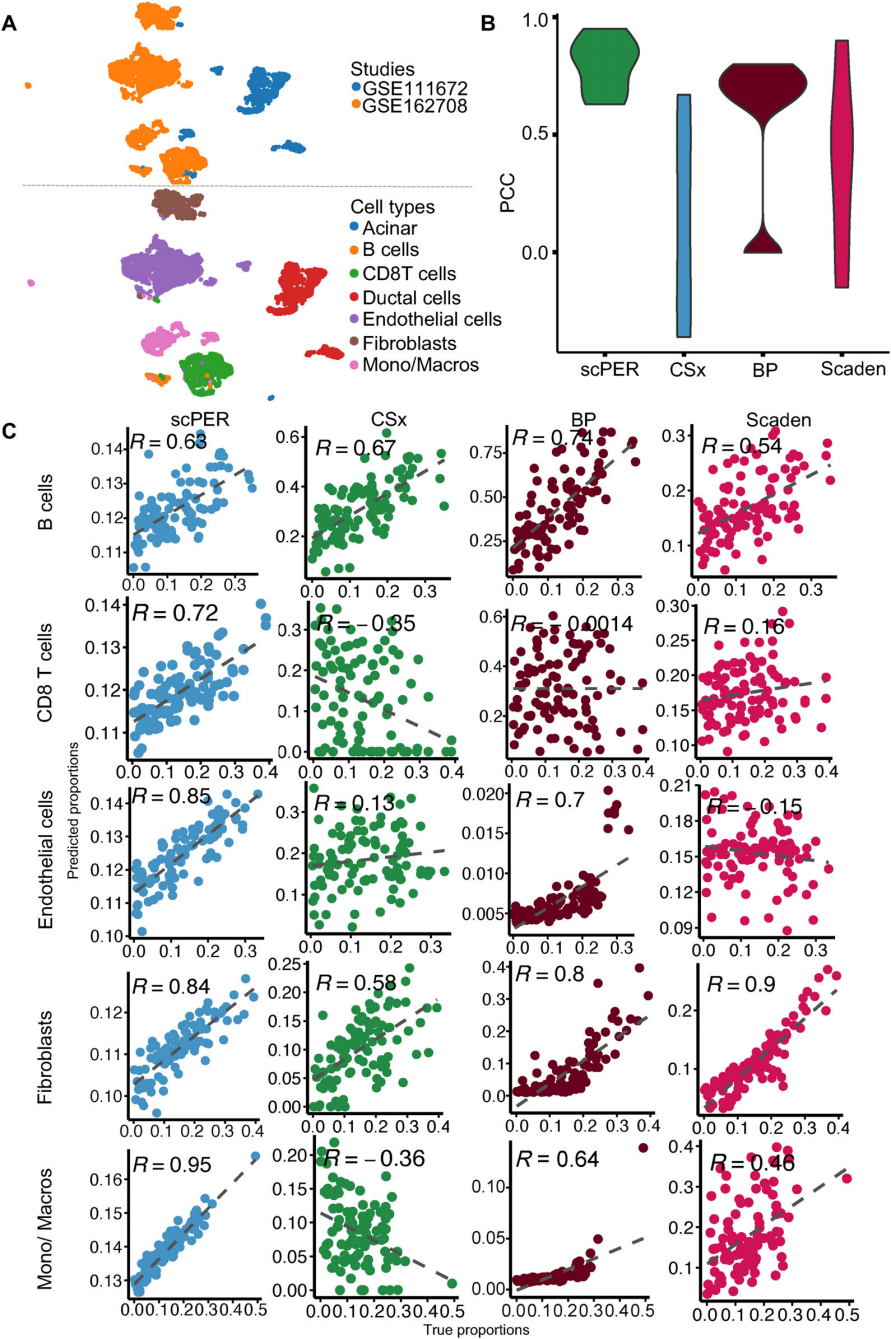
Benchmarking deconvolution across pancreatic cancer scRNA‑seq cohorts. (A) Uniform Manifold Approximation and Projection (UMAP) of combined cells from two human pancreatic cancer studies, shown prior to batch correction. Top: cells colored by dataset of origin; Bottom: cells colored by annotated cell type from the original studies. (B) Pearson correlation coefficients between true and predicted cell‑type fractions for each deconvolution method. Higher values indicate better concordance. (C) True versus predicted proportion scatter plots for five major cell populations in the tumor microenvironment (B cells, CD8 T cells, endothelial cells, fibroblasts, and mono/macros) across all methods. Points represent individual bulk samples, n=100; the x‑axis shows simulated true fractions, and the y‑axis shows fractions predicted by each tool. Mono/Macros represents monocytes/macrophages.

We collected two human pancreatic cancer scRNA‐seq datasets^[^ ^32^, ^33^ ^]^ comprising a total of 28,255 cells. Without batch correction or integration, these two datasets exhibit significant batch effects, evidenced by the identical cell types, such as monocytes/macrophages (Mono/Macros), being segregated into two distinct clusters (**Figure** **3**A). We selected the 5,000 most variable genes from these cells to construct a comprehensive pancreatic cancer reference panel encompassing all key cell types. We designated the study ID as a confounder in scPER to minimize the batch effects existing in the latent space caused by technical or other variations present between these two studies. The 100 batch‐corrected latent dimensions effectively distinguish the seven cell types while preserving the biological variability of cells from different studies (Figure S3A, Supporting Information). We then simulated 100 pancreatic cancer bulk samples using scRNA‐seq to evaluate the deconvolution performance.^[^ ^34^ ^]^ To benchmark scPER, we calculated PCC and MSE between true and predicted cell‑type fractions, comparing its performance against CIBERSORTx, BayesPrism, and Scaden. We excluded MuSiC, SCDC, DeSide and ReCIDE from this and subsequent comparisons because they require consistent cell type presence in the reference dataset,^[^ ^21^, ^22^, ^27^, ^28^ ^]^ rendering them incompatible with our study design. scPER achieved the highest average PCC (0.76), substantially outperforming CIBERSORTx (0.13), BayesPrism (0.58), and Scaden (0.38) (Figure 3B; Table S2, Supporting Information). Consistent with this, scPER also yielded the lowest MSE and mAD across cell types (Figure S3B,C, Supporting Information), indicating more accurate and stable deconvolution of pancreatic cancer bulk samples than the other methods. A closer examination per cell type revealed particularly strong gains for CD8 T cells, endothelial cells, and mono/macros (Figure 3C). Notably, endothelial cells and mono/macros were the two populations most affected by inter‐study batch effects prior to correction (Figure 3A), and scPER delivered dramatic improvements in mitigating these biases. Together, these results demonstrate the capacity of scPER to integrate multiple scRNA‐seq datasets into a unified reference panel and to accurately deconvolute cell‐type proportions in bulk RNA‐seq samples.

### 2.4 Solid Estimation of Tumor Microenvironment Components Across Tissues

**Figure 4:**
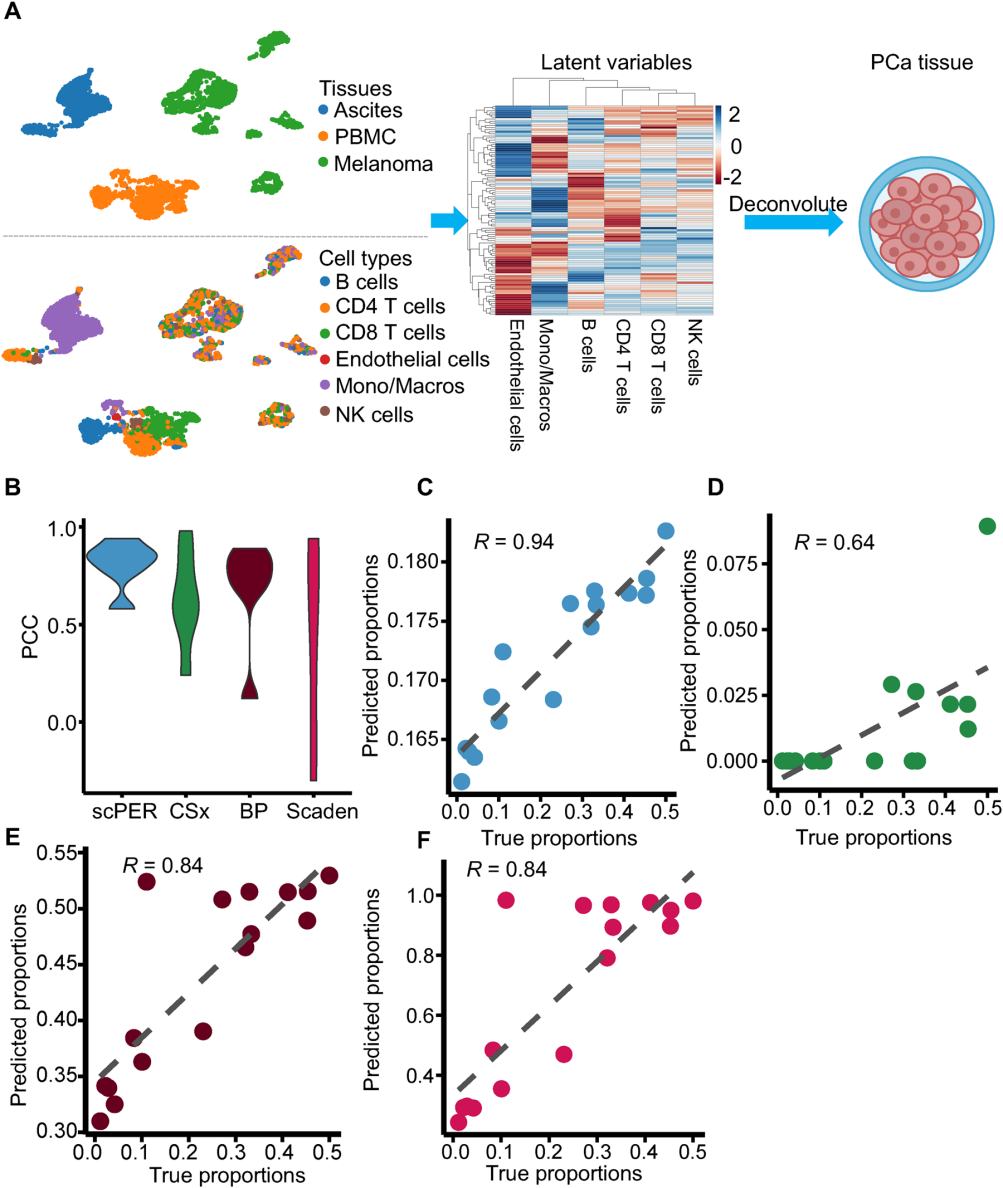
Cross‑tissue deconvolution performance comparison. (A) Workflow and latent embedding of the cross‑tissue reference panel. Left: UMAP of single‑cell transcriptomes from melanoma, ovarian cancer ascites, and PBMC sources before batch correction. Top subpanel: cells colored by tissue of origin; bottom subpanel: cells colored by annotated cell type. Middle: heatmap of scPER's 100 latent representations after adversarial batch correction, with rows as embedding features and columns as six cell types. (B) Pearson correlation coefficients between true and predicted cell‑type fractions for each deconvolution tool, summarizing overall accuracy across the 16 simulated prostate cancer bulk samples. (C‐F) True versus predicted proportion scatter plots for monocytes/macrophages using (C) scPER, (D) CIBERSORTx, (E) BayesPrism, and (F) Scaden. Points represent individual samples; the x‑axis shows ground‑truth fractions (from summed single‑cell counts), and the y‑axis shows model predictions.

Beyond correcting inter‐study variability within a single tissue, scPER also enables cross‐tissue cell‐type estimation, particularly valuable when bulk samples lack matched scRNA‑seq references. To illustrate this, we built a cross‐tissue reference panel by integrating scRNA‑seq profiles of stromal and immune cells from melanoma and ovarian cancer (ascites) patients, together with PBMCs^35^ for deconvoluting prostate cancer bulk samples (**Figure** **4**A). Before batch correction, identical cell types (e.g., CD4 T cells, CD8 T cells, monocytes/macrophages) clustered separately by tissue origin (left panel, Figure 4A).

For benchmarking, we obtained scRNA‑seq data from 16 prostate cancer patients^[^ ^36^ ^]^ and simulated bulk RNA‐seq samples by summing read counts across all cells per patient (Figure 4A). Treating tissue type (ascites, melanoma, PBMC) as a confounder, scPER applied adversarial deconfounding during training to harmonize the latent space. We then used scPER, CIBERSORTx, BayesPrism, and Scaden to predict tumor microenvironment cell proportions and computed Pearson correlation coefficients between predicted and true fractions. scPER outperformed all other methods, achieving the highest average PCC and the lowest MSE and mAD across cell types (Figures 4B; S4A,B and Table S2, Supporting Information). The error distributions for CIBERSORTx and BayesPrism were notably broader and shifted upward, whereas scPER and Scaden showed tighter, lower‐error profiles. The largest performance gains for scPER were observed in monocyte/macrophage deconvolution (Figures 4C–F; S4C, Supporting Information).

### 2.5 Proportions Estimated by scPER can Predict the Response to Anti‐PD‐1 Therapy in Patients with Metastatic Melanoma

Immune checkpoint blockade (ICB) has been approved for treating a broad range of cancer types and has shown longer‐lasting treatment benefits.^[^ ^37^, ^38^ ^]^ However, the major challenge is that only a small portion of patients respond to the ICB treatment (≈30% in solid tumors).^[^ ^39^ ^]^ To stratify the patients according to the potential response rate prior to treatment, we employed the scPER to estimate the proportions of cells in the tumor microenvironment for metastatic melanoma patients and utilized the estimated compositions to predict the response rate.

**Figure 5:**
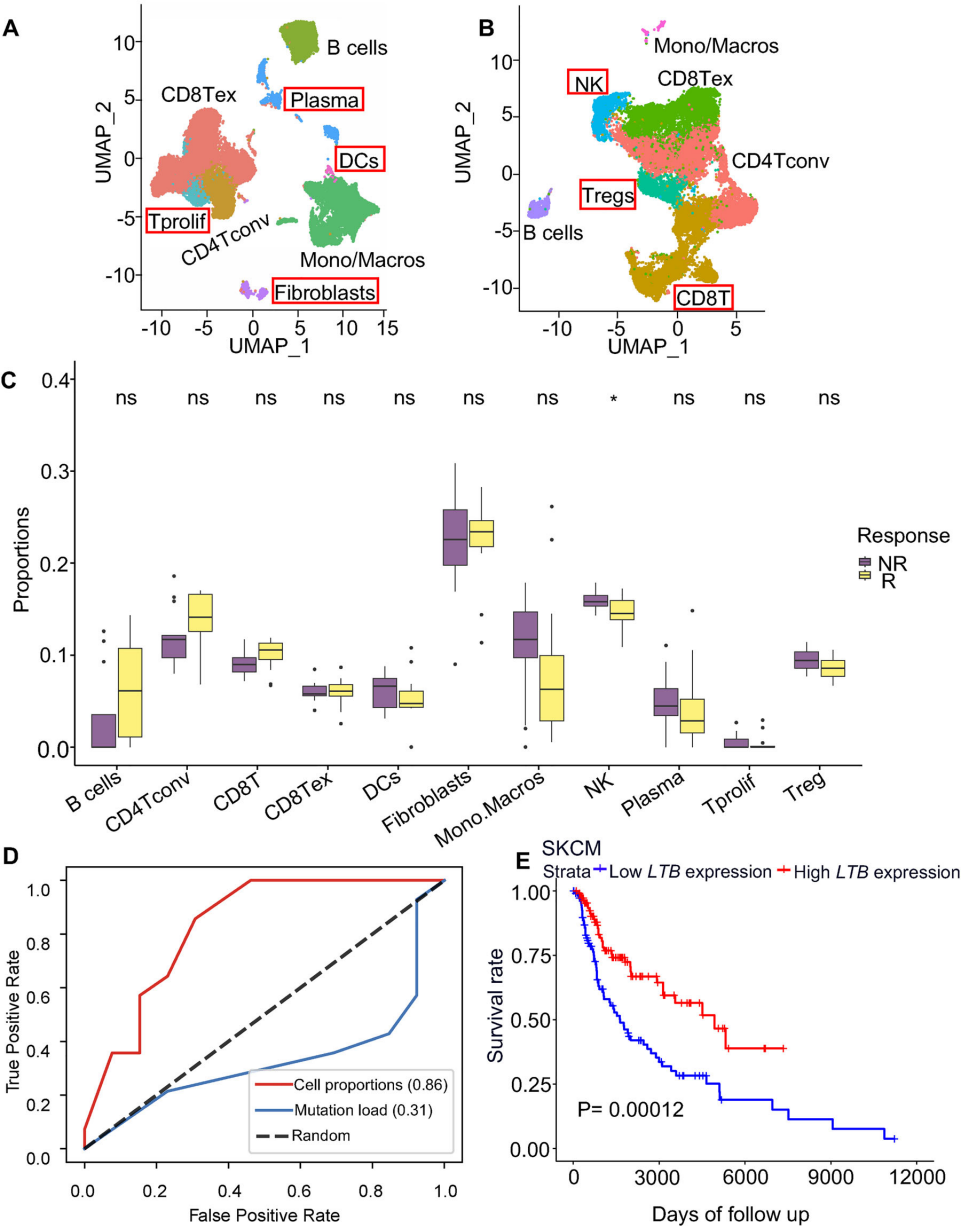
scPER‐derived cell‐type proportions predict melanoma immunotherapy outcomes. (A‐B) UMAP projections of scRNA‐seq from two melanoma datasets before scPER integration. Colored by annotated cell types. Red rectangles highlight cell populations unique to one study.(C) Boxplots comparing estimated proportions of each cell type between responders (R; n=14) and non‐responders (NR; n = 13) to immunotherapy. P‐values were calculated using the Wilcoxon rank‐sum test. (D) Receiver operating characteristic (ROC) curves for XGBoost classifier predicting response status: one using scPER‐estimated cell‐type proportion features alone and another using tumor mutation burden (mutation load) alone. Area under the ROC curve (AUROC) values are indicated for each model. (E) Kaplan‐Meier survival analysis of *LTB* expression in the TCGA‐SKCM cohort. Patients were stratified into high‐ and low‐*LTB* expression groups based on the median; the log‐rank test p‐value is shown.

We curated two scRNA‑seq datasets^[^ ^40^, ^41^ ^]^ encompassing complementary cell types, some populations appearing in only one study (highlighted in red rectangle; **Figure** **5**A,B). We then applied scPER to harmonize these datasets and attenuate technical biases, resulting in a unified reference panel of 11 cell types. Next, we used this integrated panel to deconvolute bulk RNA‑seq profiles from 27 pre‑treatment metastatic melanoma patients.^[^ ^42^ ^]^ Some cell types increased in composition in the response groups, while some decreased, with NK cells displaying a significant decrease in the response groups (Figure 5C). We next sought to investigate whether the proportions of these cell types could be used to classify the response and non‐response patients to the anti‐PD‐1 therapy. To do this, we built an XGBoost classifier and used the predicted proportions as features to classify the response and non‐response patients. We first performed the feature selection process using the mutual information score and selected the proportions of conventional CD4 T cells (CD4Tconv) and monocytes/macrophages (Mono/Macro) as the final features for the classification model (Table S3, Supporting Information). The model demonstrated improved performance with an AUROC of 0.86 when utilizing the proportions of the two cell types as features, compared to utilizing the mutation load feature (Figure 5D). For comparison, we repeated the same process with cell fractions derived from CIBERSORTx, Scaden, and BayesPrism. scPER‐derived proportions yielded the strongest predictive performance (AUROC 0.86) versus CIBERSORTx (0.68), Scaden (0.46), BayesPrism (0.41), and even a model using the top 10 mutual‐information ranked genes (0.80) (Figure S5A, Supporting Information).

We further investigated the biological mechanisms of conventional CD4 T cells in modulating immunotherapy responses for melanoma patients. We examined the top 50 marker genes of conventional CD4 T cells in both scRNA‐seq datasets and found lymphotoxin beta (*LTB*) was consistently identified as the significantly highly expressed gene for the conventional CD4 T cells in both datasets (Tables S4 and Figure S5, Supporting Information). We subsequently performed the survival analysis of *LTB* in the TCGA Skin Cutaneous Melanoma (SKCM) cohort and found that it significantly increased the survival probability (Figure 5E). Collectively, our data support that the survival improvement associated with high *LTB* expression is, at least in part, attributable to the increased abundance of conventional CD4 T cells.

### 2.6 Dissecting the T Cell Population Suggested That TGFβ Inhibits CD4 Naïve Cells to Attenuate the Response to PD‐L1 Blockade Therapy in Patients with Metastatic Urothelial Cancer

**Figure 6:**
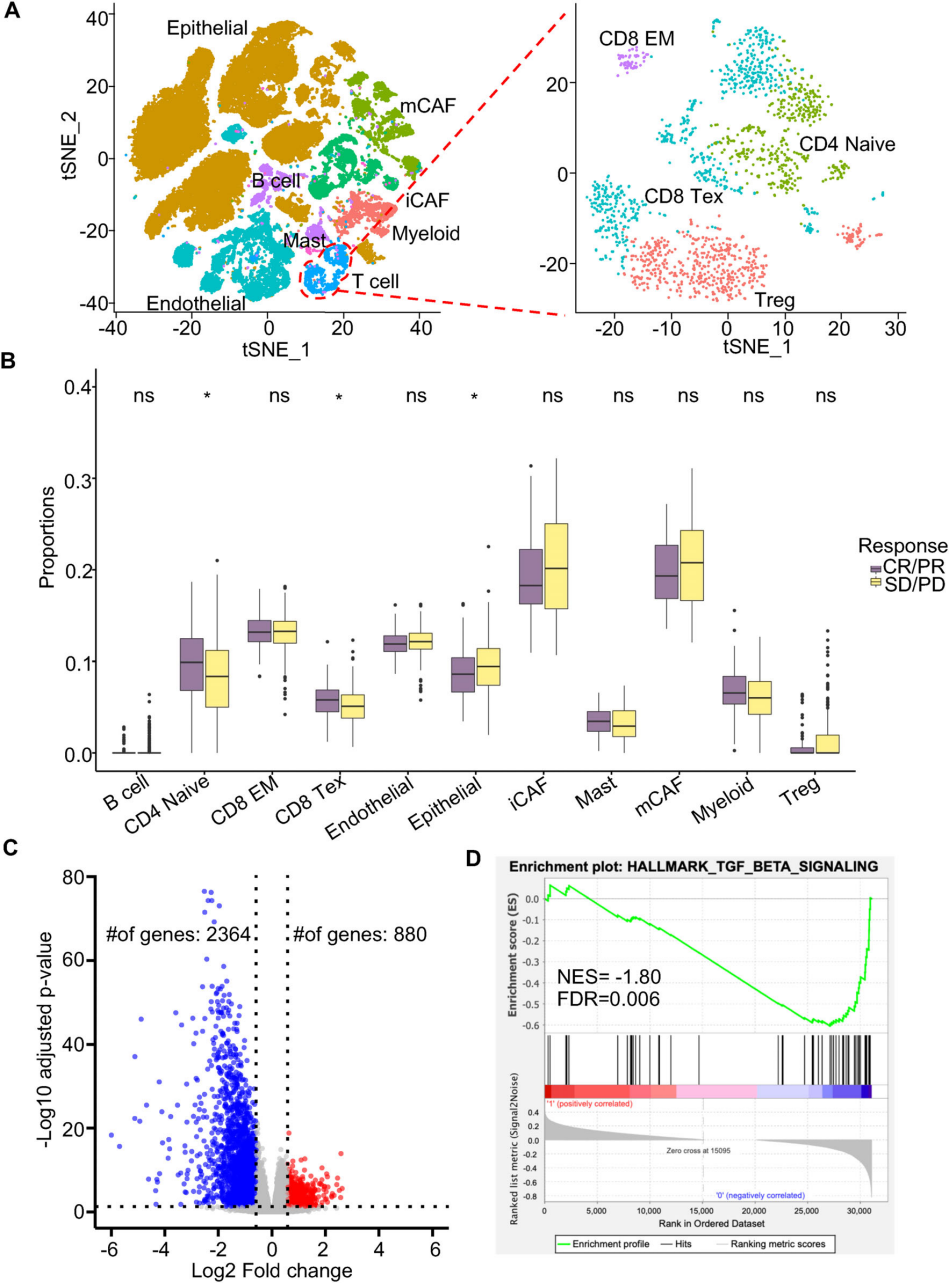
scPER reveals a key role for CD4⁺ naïve T cells in immunotherapy response. (A) t‑SNE projection of single cells from bladder urothelial carcinoma samples. The left panel includes all cell types and the right panel includes the subclusters of T cells. (B) Boxplots showing scPER‑estimated proportions of T cell subtypes and other cell types in patients who responded (n=68) versus did not respond to atezolizumab (n=230). P‐values were calculated by the Wilcoxon rank‑sum test. (C) Volcano plot of differential gene expression between patients with high versus low CD4 naïve T cell proportions. The x‑axis shows log_2_ fold change, and the y‑axis shows ‐log_10_(adjusted p‑value); genes with |log_2_FC| > 0.585 and adjusted p < 0.05 are highlighted. (D) GSEA of significantly downregulated genes from panel C (p < 0.05), with normalized enrichment scores and FDR q‑values for top pathways.

We further extended the investigation of the role of the tumor microenvironment in modulating the immunotherapy response for urothelial cancer patients. To construct the detailed reference panel to deconvolute the bulk patient samples, we collected the scRNA‐seq of 52,721 cells from eight patients diagnosed with bladder urothelial carcinoma without treatment^[^ ^43^ ^]^ (**Figure** **6**A). Here, we sought to investigate the specific role of each T cell subtype. Therefore, we subclustered T cells into four types, including CD8 effector memory T cells, CD8 exhausted T cells, CD4 naïve T cells and T regulatory cells (Figure 6A). We then combined these T cell subtypes with other existing major cell types in the tumor microenvironment and employed the scPER to build the reference panel for deconvolution. We collected 298 pre‐treatment patient samples with metastatic urothelial cancer (mUC) from a large phase 2 trial (IMvigor210) with known response information to PD‐L1 blockade with atezolizumab.^[^ ^44^ ^]^ After applying the scPER to estimate the proportions of TME cells with the built reference panel, we identified three critical cell populations whose proportions differed significantly between atezolizumab responders and non‑responders (Figure 6B). Of note, CD4 naïve T cells significantly increased in responders compared to non‐responders (Figure 6B). To investigate the mechanisms of CD4 naïve T cells in modulating immunotherapy response, we divided the 298 mUC patients into two groups based on the median proportion of CD4 naïve T cells. Upon comparison of gene expression between the groups, we found that many more genes (n = 2,364) significantly decreased in the high CD4 naïve T cell group compared to the low CD4 naïve T cell group (n = 880) (Figure 6C). Gene Set Enrichment Analysis (GSEA) revealed that multiple critical pathways were significantly enriched in the group with low CD4 naïve cell proportion, with particular emphasis on the TGFβ signaling pathway (Figures 6D; S5B, Supporting Information). TGFβ was well‐known to inhibit T cell proliferation, activation, and effector functions.^[^ ^44^, ^45^, ^46^ ^]^ Collectively, these findings suggested that TGFβ may attenuate the immunotherapy response by inhibiting the CD4 naïve cells in metastatic urothelial cancer patients.

### 2.7 A novel T Cell Subpopulation is Associated with an Attenuated Immunotherapy Response in Melanoma Patients

**Figure 7:**
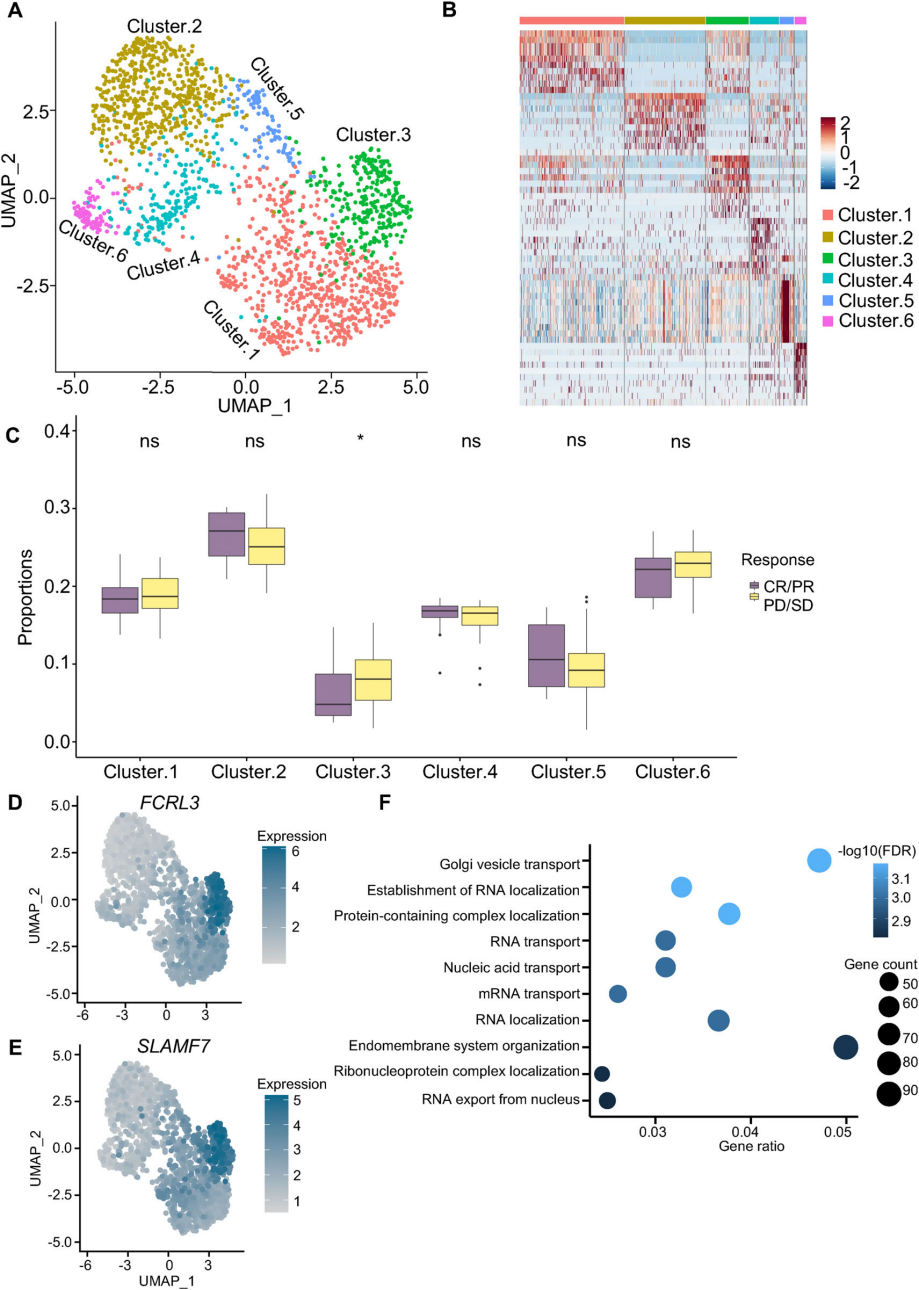
Identification of immunotherapy response‐associated T cell subclusters. (A) UMAP embedding of T cells, colored by six subcluster identities revealed by scPER subclustering. (B) Heatmap of the top 10 marker genes for each T cell subcluster, with expression values Z‐scored across cells. (C) Boxplots comparing subcluster proportions between responders and non‐responders; statistical significance assessed by the Wilcoxon test. (D‐E) UMAP feature plots showing expression of *FCRL3* (D) and *SLAMF7* (E) across the T cell subclusters; color intensity corresponds to log‐normalized expression. (F) Bar chart of enriched biological pathways among cluster 3 marker genes, including pathway name, gene ratio, gene counts, and adjusted p‐value (FDR).

In addition to estimating the proportions of predefined cell populations in bulk samples, scPER is also capable of identifying the novel phenotype‐associated subpopulations of specific cell types. Here, we focused on the 1,902 T cells from the metastatic melanoma tumor microenvironment^[^ ^47^ ^]^ and collected 104 bulk melanoma patient samples with known response information to immunotherapy from two studies.^[^ ^42^, ^48^ ^]^ We performed unsupervised clustering of the T cells and generated six subclusters (**Figure** **7**A), which displayed distinct transcriptomic profiles (Figure 7B). Subsequently, we constructed a reference panel of these T cells and applied it to the 104 bulk melanoma patient samples. By comparing the proportion of T cell subclusters between the responders and non‐responders, we identified that cluster 3 was significantly increased in the non‐responders (Figure 7C). Upon examining the markers of cluster 3, we discovered that *FCRL3* and *SLAMF7* were significantly highly expressed in this cluster (Figures 7D,E). *FCRL3* is a well‐known marker of regulatory T cells that can suppress the immune response of cancer patients.^[^ ^49^ ^]^ *SLAMF7* was reported to be correlated with T cell exhaustion, which can lead to immune evasion.^[^ ^50^, ^51^, ^52^ ^]^ In addition, the gene ontology enrichment analysis suggested that the upregulated genes of this cluster were related to RNA transport and localization regarding the biological processes (Figure 7F). Overall, our results demonstrated that scPER is capable of defining the biologically informative subclusters associated with the clinical phenotype.

## 3 Discussion

In this study, we present scPER as a novel scalable deep learning‐based approach for detailed dissecting bulk tissues with superior performance and enabling in‐depth analysis to identify novel subclusters and genes associated with the clinical phenotypes for evaluating mechanisms of cancer. The distinction between our work with previous studies includes integrating the transcriptomic profiles of single cells from various studies of the same tissue and different tissues by deconfounding the biological and technical variants in the latent space and constructing the reference panel with the cell types we are interested in (Figure S6, Supporting Information). In addition to estimating the cell fractions in the bulk tissue, scPER also enabled us to explore the biological interpretation of the composition changes of the major cell types during disease progression and further identify their clinical phenotype‐related subclusters. In our analysis of peripheral blood, pancreatic cancers, melanoma cancers, and urothelial cancers, scPER delivers accurate portraits of human tissues with a reference panel derived from diverse sources.

Researchers are currently dedicated to generating comprehensive cell atlases.^[^ ^53^, ^54^ ^]^ This is becoming increasingly important as technology advances and allows for more data generation and the ability to combine different types of scRNA‐seq data,^[^ ^55^, ^56^ ^]^ especially for limited or difficult‐to‐study tissue samples. While many studies have performed large‐scale sequencing of the cells across tissues, they typically focused on individual cell types, such as T cells,^[^ ^57^, ^58^ ^]^ NK cells^[^ ^59^ ^]^ and myeloid cells,^[^ ^60^ ^]^ which is not ideal for constructing reference panels, as we need to include all necessary cell types to mimic the real compositions of the tumor microenvironment. Therefore, we selected the studies that included a broader range of cell types and integrated them to construct a comprehensive reference panel. Our analysis, which involved randomly selecting 5,000 cells from various studies and integrating them by removing the batch effects for both healthy and neoplastic tissues, demonstrated that single‐cell reference profiles can provide detailed portraits of tissue composition, and inter‐subject heterogeneity did not significantly impact the results. The advantages of integrating various scRNA‐seq data for constructing reference panels include the ability to customize the signature matrices for various tissues with detailed cell types and subtypes without the need for complicated techniques such as antibody panels or cell sorting and to study the poorly understood tissues by using the integrated signature matrices from other tissues. Although cross‐tissue reference panels are not the default in deconvolution workflows, they become necessary when matched scRNA‐seq data are unavailable or incomplete, particularly for rare or difficult‐to‐dissociate tissues. In studies of metastasis or cancers of unknown primary, cross‐tissue references can also recover shared stromal and immune compartments, thereby providing a more complete view of the tumor microenvironment. By integrating diverse scRNA‐seq datasets while attenuating tissue‐ and batch‐specific confounders, scPER is well‐suited to these challenging yet clinically relevant scenarios.

To evaluate deconvolution under controlled conditions, we generated simulated bulk RNA‐seq using two complementary strategies. One drew random cell‐type proportions to mimic heterogeneous tissue compositions; the other aggregated cells using patient‐specific proportions to preserve biological realism while acknowledging possible dissociation or capture biases. Although scRNA‐seq‐based simulations cannot capture every facet of bulk transcriptomes, aggregation over large cell pools and validation on real PBMC bulk data with matched flow cytometry support the robustness of scPER across both synthetic and real‐world settings.

scPER also enables the biological association analysis between cell composition changes with the phenotypes of the bulk tissues, especially for investigating the mechanisms of immunotherapy response of patients. In our analysis of the metastatic melanoma patient samples, we demonstrated that we could employ the cell proportions estimated by scPER to predict the immunotherapy response of the patients with high accuracy (AUROC = 0.86), which significantly outperformed the accuracy using tumor mutation load. In addition, scPER also had the ability to identify the T cell subtypes (CD4 naïve cells) significantly associated with the immunotherapy response of metastatic urothelial cancer patients and suggested the critical role of the TGFβ signaling pathway in mediating the response of patients, which is consistent with the findings in the original study reporting the bulk patient samples we used.^[^ ^44^ ^]^ Moreover, scPER can also identify the novel phenotype‐associated T cell clusters that were significantly increased in PD/SD melanoma patients to the immunotherapy. By examining the highly expressed genes in this cluster, we found two critical genes *FCRL3* and *SLAMF7*. Targeting the *FCRL3* on regulatory T cells can inhibit the regulatory T cell function in suppressing the immune response.^[^ ^49^ ^]^ More importantly, *SLAMF7* was reported to be highly expressed on the immunosuppressive Tregs and correlated with T cell exhaustion.^[^ ^50^, ^51^ ^]^ However, while our computational analyses suggest that *FCRL3* and *SLAMF7* may be associated with melanoma and its immunotherapy response, further functional studies are necessary to elucidate their precise roles in melanoma biology. Future investigations, including in vitro and in vivo experiments, will be required to validate these findings and to determine the mechanistic significance of these markers in melanoma.

A core assumption of scPER is that bulk RNA‐seq can be embedded into the same latent space learned from single‐cell data because bulk profiles approximate mixtures of single‐cell states. Our PBMC and simulated benchmarks support this assumption empirically. Nonetheless, projection accuracy may decline when important cell types are absent from the reference or when platform and quality differences are extreme. The preprocessing and adversarial deconfounding of scPER help attenuate these effects, and future versions will incorporate domain‐adaptation calibration and input diagnostics to further safeguard cross‐modality embedding.

In summary, scPER is a versatile platform that can effectively dissect cellular heterogeneity in intricate tissues. It can be used to determine the proportions of desired cell types in bulk tissues, such as fixed samples from clinical trials, with or without scRNA‐seq data from the same tissue, and systematically investigate the biological associations between cell proportions with phenotypes, especially for the immunotherapy response. It can also identify specific cell subsets and novel clusters associated with the phenotypes. Employing this approach can enhance the statistical power of biological discoveries and present opportunities for integration with other techniques. Overall, scPER has the capacity to advance the analysis of multicellular systems in various organisms, including humans and mice.

## 4 Experimental Section

### Datasets and Preprocessing—Single Cell RNA‐seq Datasets

In this study, the PBMC scRNA‐seq dataset in Figure 2 was downloaded from the 10X Genomics data download page: 8k PBMCs from a healthy donor (https://www.10xgenomics.com/resources/datasets/8‐k‐pbm‐cs‐from‐a‐healthy‐donor‐2‐standard‐2‐1‐0). In Figure 3, the scRNA‐seq data of pancreatic cancers used for simulating bulk samples and building reference panels were downloaded from the TISCH2 database.^[^ ^61^ ^]^ For Figure 4, the scRNA‐seq datasets of melanoma, ovarian cancer and PBMC were obtained from https://figshare.com as provided by Schelker et al.^[^ ^35^ ^]^ The scRNA‐seq data of prostate cancer was downloaded from the TISCH2 database. In Figure 5, the two scRNA‐seq datasets of melanoma patients were obtained from the TISCH2 database and they are metastatic tumors without any treatment. The bladder cancer scRNA‐seq dataset in Figure 6 was downloaded from the original study^[^ ^43^ ^]^ and the samples were sequenced before treatment. In Figure 7, the scRNA‐seq data of metastatic patients without treatment was downloaded from the TISCH2 database.

### Inputs Processing

Let $X\;\in R_{\geq 0}^{G\times N}$ be the raw scRNA‐seq count matrix (genes *g* = 1, …, *G*; cells *j* = 1, …,*N*) for a given tissue or study, and let $U\;\in R_{\geq 0}^{G\times N}$ be the raw bulk RNA‐seq count matrix (samples *m* = 1,…, *M*). For each cell *j*, let *b_j_* and  *t_j_* denote its batch and tissue labels when applicable. All downstream analyses use the common gene sets G* = genes (*X*) ∩ genes (*U*).

### scRNA‐seq Preprocessing and Analysis

The R package Seurat (v. 4.0.1)^[^ ^62^ ^]^ was utilized to process all scRNA‐seq datasets following the typical workflow. Initially, we filtered the expression matrices for cells with fewer than 200 detected genes and genes expressed in less than three cells. Let  *d_j_* = $\sum_{g}1\left\{\;X_{gj}>0\right\}$. Outlier cells with extreme  *d_j_* values were excluded using a quantile rule ([*Q*_0.01_, *Q*_0.99_]) applied to each dataset.

Seurat function “NormalizeData” was used to normalize the gene expression to library size. For each cell *j*, define its library size  *L_j_* = $\sum_{g\;}X_{gj}$. We computed counts‐per‐10k

(1) $$Y_{gj}=10^{4}\cdot \frac{\;X_{gj}}{\;L_{j}}\;$$

### And Applied to a Log1p Transform

(2) $$\tilde{Y_{gj}}=\log\left(1+Y_{gj}\right)$$

Louvain clustering implemented in the Seurat package was used to cluster all cells, and the top 2,000 highly variable genes were used for clustering, which was calculated using Seurat's variance‐stabilizing (“vst”) method. Let $\bar{\mu }$ *_g_* and $\bar{\upsilon }$ *_g_* denote the mean and variance of $\tilde{Y_{g}}$ across cells; the standardized dispersion is

(3) $$\delta_{g}=\log{\bar{\upsilon }}_{g}-f\;\left(\log{\bar{\mu }}_{g}\right),$$

where *f*(·) is a smooth mean‐variance trend. This was retained the top 2,000 genes for graph clustering.

Principal components analysis was employed for dimensionality reduction. We defined the cell types using the markers from the original study and ensured that the labeling of cell types remained consistent across all datasets pertaining to a particular tissue (Figures S7A—G, Supporting Information). For PBMC dataset, examples include dendritic cells (*FCER1A, CLEC4C, IL3RA*), NK cells (*NKG7, GNLY, KLRD1*; *CD3D/CD3E* negative), B cells (*MS4A1, CD79A, CD79B*), CD4 T cells (*IL7R, CCR7*), CD8 T cells (*CD8A, GZMB*) (FigureS7A, Supporting Information). For pancreatic cancer datasets, acinar cells (*PRSS1, PRSS3, CPA1*), ductal cells (*KRT8, KRT18, KRT19*), endothelial cells (*PECAM1, VWF, FLT1*), fibroblasts (*COL1A1, COL1A2, COL3A1*) were used (FigureS7B, Supporting Information). The markers we used to define the cell types for cross‐tissue (FigureS7C), two melanoma datasets (FigureS7D,E, Supporting Information) and bladder cancers (FigureS7F, G, Supporting Information) are likewise provided.

### Tissue Datasets for Benchmarking and Exploring the Roles of Proportion Changes in Response to Immunotherapy

In order to evaluate the performance of deconvolution tools on real tissue expression data, the bulk RNA‐seq data was obtained of 164 pre‐ and post‐vaccination human PBMC samples with flow cytometry‐measured cell proportions.^[^ ^30^ ^]^ The bulk pre‐treatment patient samples of melanoma^[^ ^42^, ^48^ ^]^ and urothelial cancer^[^ ^44^ ^]^ were downloaded from the original studies.

### Simulation of Bulk RNA‐seq Samples Using scRNA‐seq Data

To comprehensively evaluate the performance of the deconvolution approaches, this was also simulated the various bulk RNA‐seq samples using the scRNA‐seq data. We employed two different simulation strategies depending on the patient sample size of the scRNA‐seq datasets.

The first simulation strategy is based on the calculated cell proportions in the scRNA‐seq dataset, proposed by a previous study ^[^ ^27^ ^]^ and widely used in benchmarking deconvolution tools. This approach was used to simulate the prostate cancer bulk samples. The scRNA‐seq data includes 16 patient samples and we simulated the bulk samples based on the true proportions of the cell types measured in the scRNA‐seq datasets, where the *f_c_* can be directly obtained from the scRNA‐seq data. Then we summed up the gene counts of all cells for each patient sample to construct the simulated bulk RNA‐seq data. While this approach has limitations, as not all cell types are equally captured or survive dissociation, it provides a standardized way to create bulk samples for benchmarking. To address the concerns of the first approach, we proposed another simulation method for pancreatic tumors. Instead of using calculated proportions from the scRNA‐seq data,

This was randomly simulated the proportions for each cell type in the bulk samples and then selected a certain number of cells for each type based on these simulated proportions. Specifically, a random number was selected from a uniform distribution of 0 to 1 for each cell type using R (v 4.2.1) function “runif”. To ensure that the proportions added up to 1, the number was then divided by the sum of all generated random numbers

(4) $$f_{c}=\frac{r_{c}}{\sum_{C_{all}}r_{c}}$$

where *r_c_* represents the random number generated for cell type *c* and *C_all_* refers to the collection of all cell types. Here, *f_c_* represents the computed proportion for cell type *c*. The fractions were then multiplied by the total number of cells that were chosen in the scRNA‐seq data, ultimately yielding the number of cells to be selected for each cell type

(5) $$N_{c}=f_{c}*\;N_{total}\;$$

where *N_c_* represents the number of cells to be selected for cell type *c* and *N_total_* represents the total number of number cells in the selected scRNA‐seq data. Then, for each cell type *c*, *N_c_* cells were randomly selected from the single cell expression matrix. Next, the single‐cell expression profiles that were selected at random for each cell type were combined by adding their expression values to produce the expression profile for the simulated bulk sample. This process was repeated 100 times to generate 100 simulated bulk samples for pancreatic cancers. This method aims to mimic more realistic scenarios where cell type proportions are not strictly tied to scRNA‐seq distributions.

We quantified gene coverage in the simulated bulk samples using these two simulation strategies. Under the proportion‐driven scheme, 16 prostate cancer simulations contained ≈16,783–20,210 detected genes (FigureS8A, Supporting Information). Under the random‐proportion scheme, 100 simulated pancreatic cancer samples contained ≈13,758–13,787 detected genes (FigureS8B, Supporting Information). These ranges are in line with typical bulk RNA‐seq libraries, indicating that aggregation recovers broad gene coverage despite single‐cell sparsity.

### Overview of the scPER Analytical Framework

### Overview of the scPER Analytical Framework—Input Data Processing

The goal of the data preprocessing step is to make the input data more suitable for scPER. To construct the reference panel of cell types, we need to select a certain number of cells from the whole scRNA‐seq dataset. We performed experiments using different numbers of cells for training and found that using 5,000 cells generally yields the strongest performance while remaining efficient. We therefore subsampled 5,000 cells for each task, balancing the per cell‐type counts via stratified sampling. We then intersected genes shared by the scRNA‐seq and bulk datasets. To evaluate the optimal number of genes for inclusion in the model, we tested feature sets of the top 3,000, 5,000, and 7,000 most variable genes in both the PBMC and cross‐tissue tasks. Sensitivity analyses showed that 5,000 highly variable genes (HVGs) achieved the best performance, as indicated by the highest PCC between predicted and true cell‐type fractions (Figures S9A,B, Supporting Information). Across both benchmarks, 5,000 HVGs yielded the most stable and consistent deconvolution results, balancing computational efficiency with model accuracy. The variances were calculated by the “var” function in R. To recover the missing expressions of the 5,000 genes, we employed the Markov affinity‐based graph imputation of cells (MAGIC) to impute the gene expressions of those 5,000 cells with the default parameters.

### Adversarial Autoencoder

To learn the biological representations of the gene expression profiles and construct the reference panels without the out‐of‐interest sources of variation, such as batch effects and tissue biases, our strategy incorporated the adversarial deconfounding autoencoder model,^[^ ^63^ ^]^ which comprises standard autoencoder and adversary network training simultaneously. The autoencoder aims to generate the latent representations that accurately reconstruct the data and the adversary network is trained to identify the confounders from the embeddings generated by the autoencoder. Autoencoder takes the input of the expression matrix *x*. The encoder network is responsible for mapping the input space *X* to the latent embedding *Z*, and the decoder network is responsible for mapping the embedding *Z* back to the input space *X*. The autoencoder networks were optimized through the following process:

(6) $$min_{\emptyset ,\varphi }E{∥x-g_{\varphi }\left(f_{\emptyset }\left(x\right)\right)∥}_{2}^{2}$$

where Φ and φ represent the parameters of the encoder and decoder networks. The objective is to minimize the squared 2‐norm distance between the input *x* and its reconstruction.

The second network is an adversary network, which attempts to predict the confounder *C* using the latent representations generated by the encoder. Autoencoder strives to reconstruct the input data while simultaneously hindering the adversary model from predicting the confounders accurately. Conversely, the adversary model updates its parameters to increase its ability to predict the confounders from the generated latent representations.

The model will be trained in three steps: (i) To generate the embedding *Z*, we first pre‐train the autoencoder by optimizing Equation 3. (ii) The adversary model is defined as a mapping from the embedding *Z* to the confounder *C* and is optimized using the following objective:

(7) $$\mathrm{mi}n_{v}E\left[L\left(h_{\nu }\left(x\right),c\right)\right]$$

The choice of the specific loss function *L* is dependent on the type of confounder being selected, and it should be a differentiable function appropriate for that type of confounder. The adversary model is pre‐trained using this loss function to optimize its ability to predict the confounder. (iii) After the autoencoder and adversary models have been pre‐trained separately, we start their joint adversarial training. During this training, the autoencoder model is first trained while freezing the parameters of the adversary model. The optimization is performed utilizing stochastic gradient descent with the following aim:

(8) $$\mathrm{mi}n_{\emptyset ,\varphi ,\nu }E\left[∥x-g_{\varphi }\left(f_{\emptyset }\left(x\right)\right){∥}_{2}^{2}-\lambda L\left(h_{\nu }\left(x\right),c\right)\right]$$

The autoencoder model is updated to minimize Equation 6 and maximize Equation 7 by minimizing the negative of the objective. Once the autoencoder model is optimized, it is frozen, and the adversary model is trained for one epoch to minimize Equation 7. This training process is alternated until both models are optimized. The adversarial weight was fixed at *λ* = 1 in this study based on the sensitivity analysis on the pancreatic cancer deconvolution task: mean PCC across cell types for λ∈{0.5,1,2} was 0.776, 0.798 and 0.794, respectively, indicating a performance plateau ≈*λ* = 1.

### Bulk Sample Processing and Proportion Estimation

Sensitivity analyses showed that 100 latent dimensions provided the best performance, yielding the highest PCC between predicted and true cell‐type fractions (Figures S9C,D, Supporting Information). Both smaller latent sizes (80) and larger sizes (120) resulted in decreased PCC, indicating underfitting and overfitting, respectively. These results suggest that 100 latent dimensions strike the optimal balance between model complexity and deconvolution accuracy for both PBMC and cross‐tissue tasks. Accordingly, after training both the autoencoder and adversary models with the scRNA‐seq data, we extracted the 100 latent representations of each cell and calculated the median values of the 100 latent vectors for each cell type, which were then considered the representations for each cell type. Then we applied the trained models to embed the bulk tissue samples and generate the 100 latent representations for each bulk sample. Afterward, we employed the XGBoost regression model^[^ ^64^ ^]^ to estimate the coefficients of each cell type as below:

(9) T=P∗R

where *T* is the latent representation in the bulk sample, and *R* is the latent representation in the reference panel constructed using the scRNA‐seq. *P* is the estimated coefficient of each cell type in the regression model. The negative coefficients from the XGBoost regression model are then set to 0, and the remaining coefficients are normalized and assured their sum equals 1, resulting in a final vector of calculated cell type proportions.

### Benchmark with Other Deconvolution Tools

This was compared scPER with seven popular cell deconvolution approaches: CIBERSORTx, MuSiC, BayesPrism, Scaden, SCDC, DeSide and ReCIDE. These methods were chosen for their diverse methodologies: CIBERSORTx employs support vector regression, BayesPrism utilizes the Bayesian method, MuSiC uses weighted non‐negative least‐squares regression, Scaden and DeSide leverage deep neural networks and SCDC adopts the revised W‐NNLS from MuSiC and combines it with the ENSEMBLE method. ReCIDE is a framework to integrate multiple deconvolution tools. We tested these tools on both real PBMC bulk samples and various simulated bulk samples, calculating the PCC, MSE and mAD between the true and predicted proportions to evaluate their performance.

CIBERSORTx (https://cibersortx.stanford.edu/index.php) generates the gene expression signature matrices from the scRNA‐seq data and performs batch normalization between reference panels and bulk samples. However, it cannot correct the batch effects of various scRNA‐seq studies when constructing the reference panel (Figure S6, Supporting Information). We uploaded the scRNA‐seq matrix and bulk samples to the website and ran the analysis with the default options. For PBMC bulk RNA‐seq samples, we selected S‐Mode for batch normalization, while no batch normalization was applied to the simulated bulk samples.

BayesPrism utilizes the Bayesian method to predict cellular composition by constructing the reference panel from the scRNA‐seq data. We tested it in the three scenarios following their instructions (https://github.com/Danko‐Lab/BayesPrism).

MuSiC can integrate scRNA‐seq datasets from various sources for constructing reference panels by correcting the bias, but it requires that the cell types exist in all datasets (FigureS6, Supporting Information), which was not applicable to our tests in Figures 3 and 4. Therefore, we tested it only on the PBMC datasets (Figure 1) using the R package with default options.

Scaden employs multiple neural networks during the training process and selects the best models to predict cell proportions. However, it lacks the strategies to correct the batch effects in scRNA‐seq studies (Figure S6, Supporting Information). We followed the tutorial on the GitHub page (https://github.com/KevinMenden/) to train the model and predict the cell proportions for each task.

SCDC was developed to integrate deconvolution results from various scRNA‐seq reference panels. However, it requires all cell types to be present in each study, making it unsuitable for our cross‐pancreatic cancer study and cross‐cancer type analyses. Therefore, we only applied it to the PBMC data.

DeSide and ReCIDE were employed following the instructions from their GitHub repository.

### Feature Selection for Immunotherapy Response Prediction

We utilized mutual information scores to assess the significance of cell type proportions for predicting immunotherapy response, measuring how much uncertainty is reduced for one variable when another variable is known. Using the SelectKBest function from the scikit‐learn 1.1.1 Python library, we computed the mutual information score for each cell type proportion against a target label (response/non‐response). This score represents the reduction in uncertainty of one variable given the knowledge of another. We performed this calculation twenty times and averaged the results to obtain the final score for each cell type proportion (Table S3, Supporting Information). To determine the optimal number of cell type proportions for inclusion in the predictive model, we ranked the cell type proportions according to their mutual information scores and built an XGBoost classifier to evaluate the performance across different feature counts.

### Survival Analysis

We downloaded the transcriptomic profile and clinical data of TCGA SKCM from the UCSC Xena platform.^[^ ^65^ ^]^ A Univariate Cox proportional hazards model from the R package “survival” was used to fit the gene expression. To visualize the survival distributions, we employed Kaplan–Meier survival plots created by the “survfit” function from the R survival package.

### Pathway Enrichment Analysis

Gene Set Enrichment Analysis (GSEA) was conducted using GSEA 4.3.1^[^ ^66^, ^67^ ^]^ with Human MSigDB hallmark gene sets and Gene Ontology enrichment analysis was performed using WebGestalt 2019^[^ ^68^ ^]^ with a focus on the biological processes.

### Statistical Analysis

The linear concordance between true and predicted cell type proportions was measured using Pearson correlation (r). All statistical analysis was conducted using R 4.2.1 software. Significance was determined by the Wilcoxon rank‐sum test unless specified otherwise in the figure legend. Significance was considered if the p‐value was <0.05, while in pathway analysis, significance was considered if the FDR was <0.05 after correction for multiple comparisons.

## Conflict of Interest

The authors declare no conflict of interest.

## Author contributions

X.Z. and R.K. contributed equally to this work. BL, XZ, and RK contributed to conceptualization, data curation, investigation, methodology, supervision, and validation; BL additionally performed formal analysis, software development, visualization, project administration, and prepared the original draft; XZ and RK secured funding; and BL, XZ, and RK all participated in project administration, provided resources, and contributed to writing, review, and editing.

## Supporting information



Supporting Information

Supporting Information

Supporting Information

Supporting Information

Supporting Information

Supporting Information

## Data Availability

scPER is an available tool on GitHub (https://github.com/BrianLlll/scPER). The public datasets used in this study were downloaded from various data sources, including 10X Genomics (https://www.10xgenomics.com/resources/datasets/8‐k‐pbm‐cs‐from‐a‐healthy‐donor‐2‐standard‐2‐1‐0), the TISCH2 database, and figshare (https://figshare.com), are described in the Results and Methods sections.
