# Supplementary material for: scPER: A Rigorous Computational Approach to Determine Cellular Subtypes in Tumors Aligned With Cancer Phenotypes From Total RNA Sequencing
Source: Adv Sci (Weinh). 2025 Nov 27;13(8):e14502. doi: 10.1002/advs.202514502 (PMC12884779; doi:10.1002/advs.202514502)
Supplement: Supplementary file 1 — Supporting Information [file ADVS-13-e14502-s001.pdf]

Fig. S1

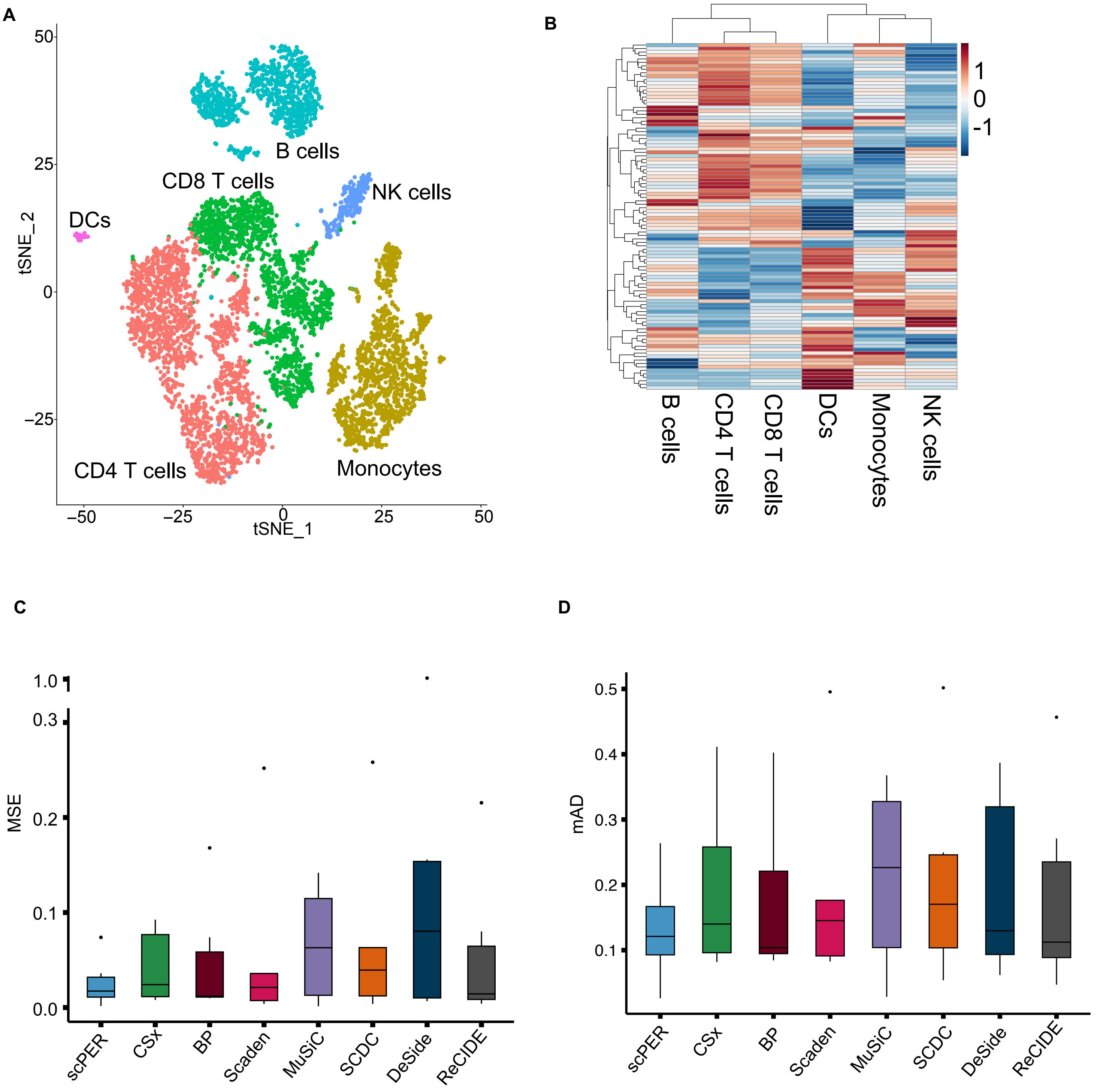

Fig. S1. Assessment of deconvolution on PBMC single-cell embeddings and bulk predictions. (A) t-Distributed Stochastic Neighbor Embedding (t-SNE) of PBMC scRNA-seq profiles based on raw gene expression, colored by annotated cell type. (**B**) Heatmap of 100-dimensional latent embeddings learned by the adversarial deconfounding autoencoder, with rows corresponding to embedding features and columns to cells grouped by cell type. (**C**) Mean squared error (MSE) **(D)** and mean absolute deviation (mAD) between true and predicted cell-type proportions for each deconvolution method, shown as bar plots. Lower values indicate more accurate deconvolution.

Fig. S2

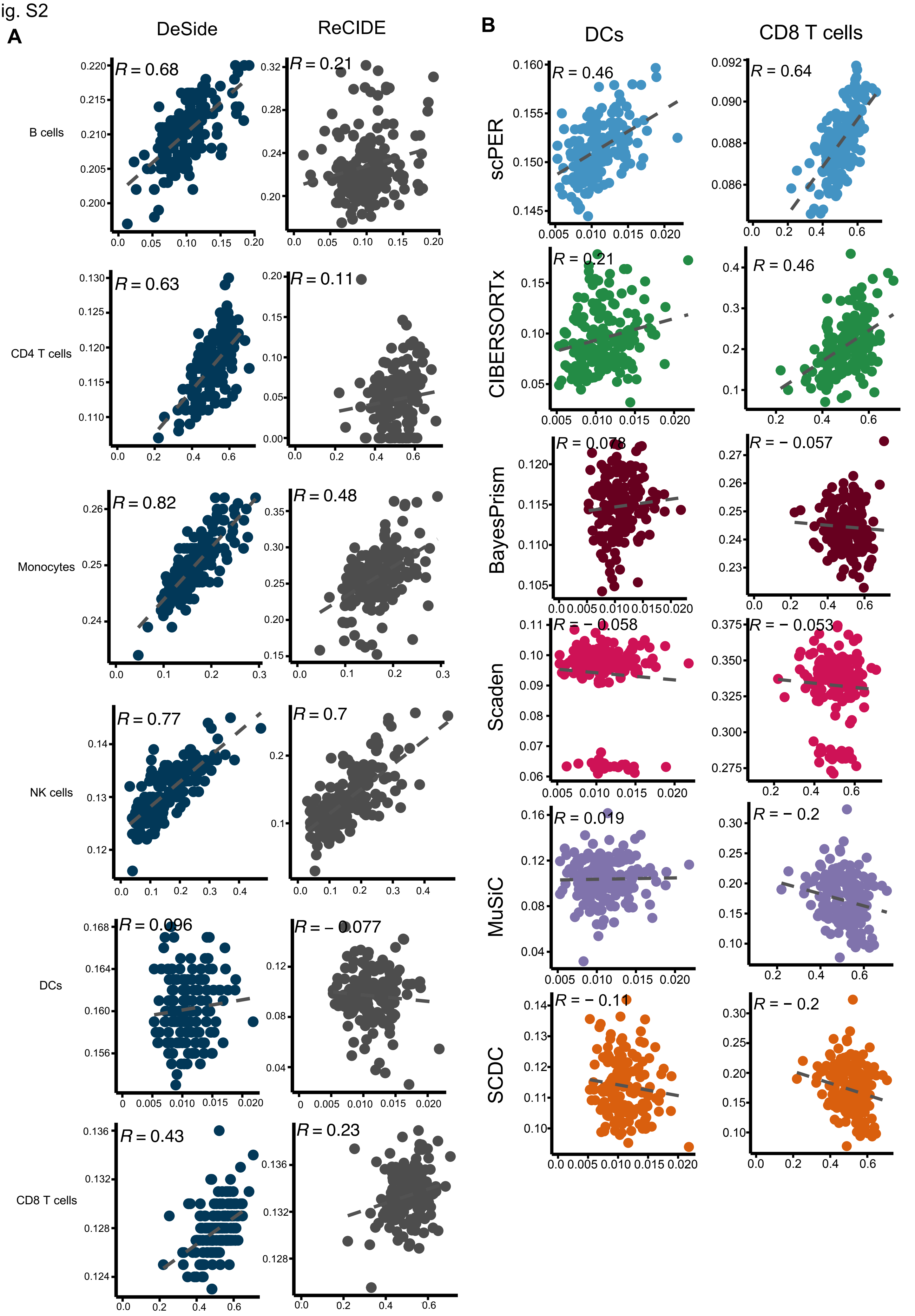

Fig. S2. Scatter plots comparing true versus predicted proportions. (**A**) All cell types for DeSide and ReCIDE. (**B**) DC and CD8 T cells for benchmarking tools. Each point represents one bulk sample; the x-axis shows ground-truth fractions (flow cytometry), and the y-axis shows model predictions.

Fig. S3

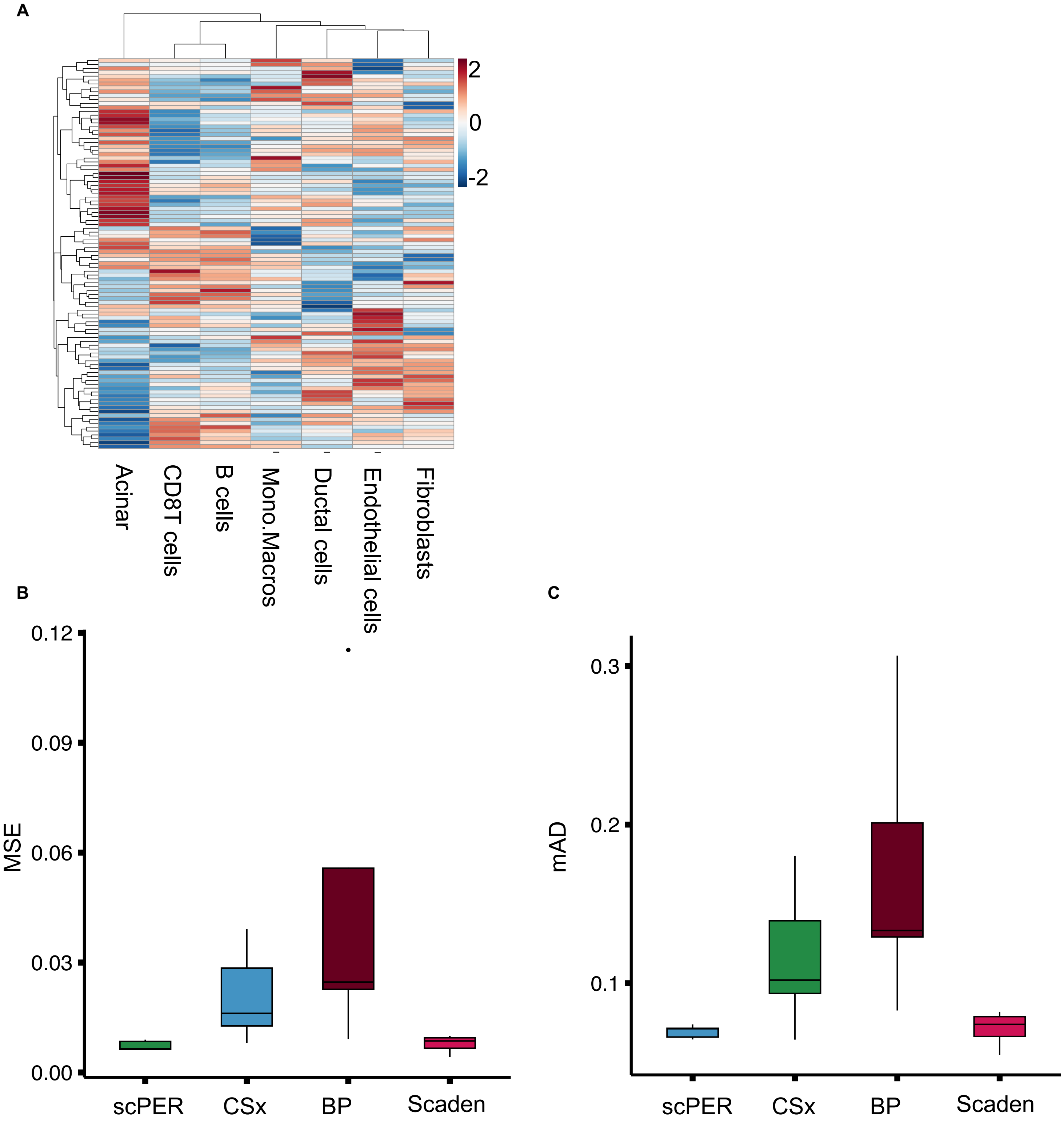

Fig. S3. Latent embedding and Error metrics for deconvolution methods. (**A**) Heatmap of the 100-dimensional latent embeddings for cells from two human pancreatic cancer scRNA-seq datasets, following adversarial deconfounding. Rows represent individual embedding dimensions; columns represent annotated cell types. Distinct blocks indicate successful batch mixing alongside cell-type-specific signal. **(B)** Bar plot of MSE and (**C**) mAD between true and predicted cell-type proportions for each deconvolution tool. Lower MSE values reflect more accurate predictions of cell-type abundance in bulk samples.

Fig. S4

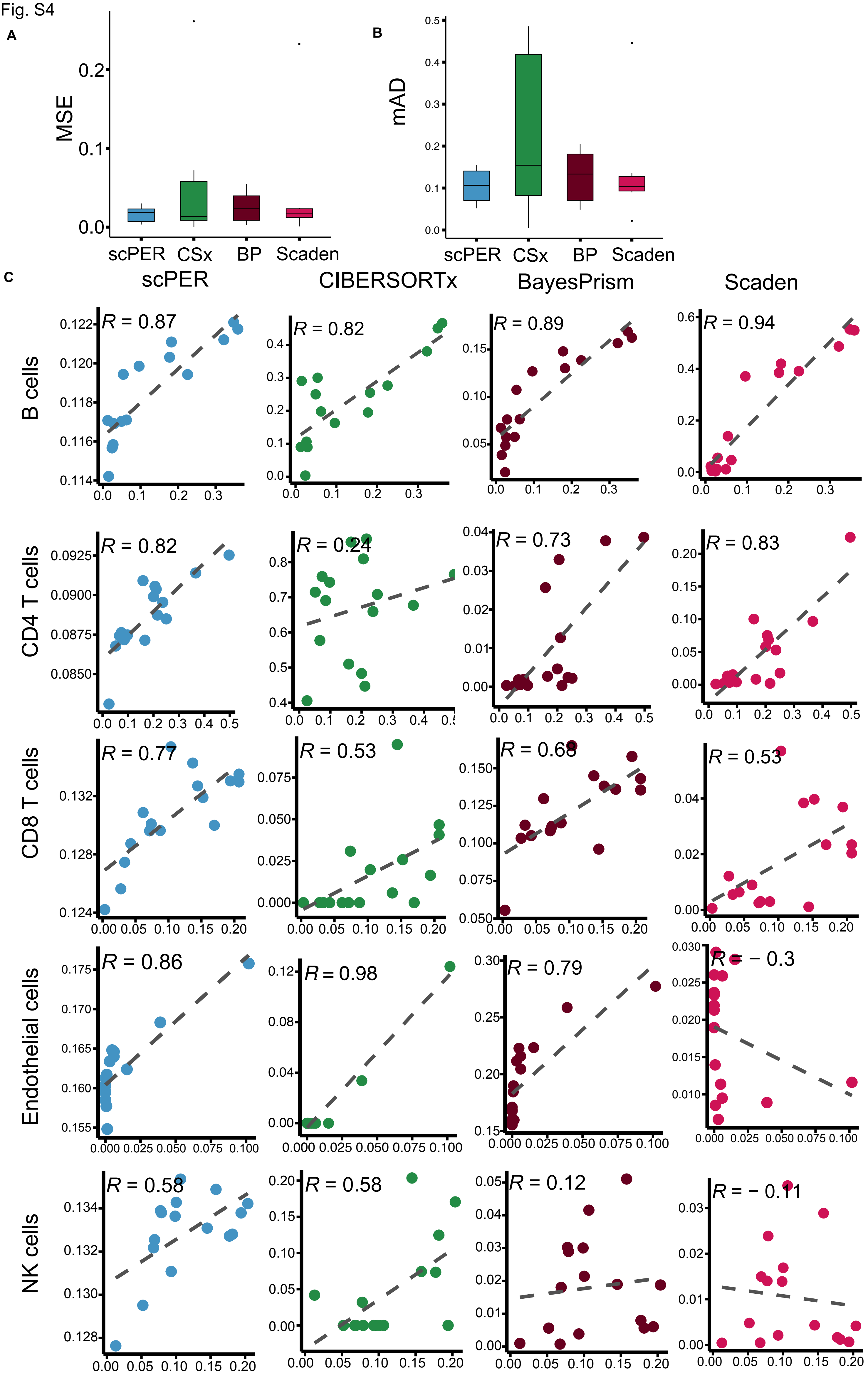

Fig. S4. (A) Mean Squared Error (**B**) mAD was calculated by comparing the true and predicted fractions for each tool. **(C)** Scatter plots of the five cell types for each tool compare the true and predicted proportions. The y-axis is the predicted proportion, while the x-axis is the true proportion.

Fig. S5

A

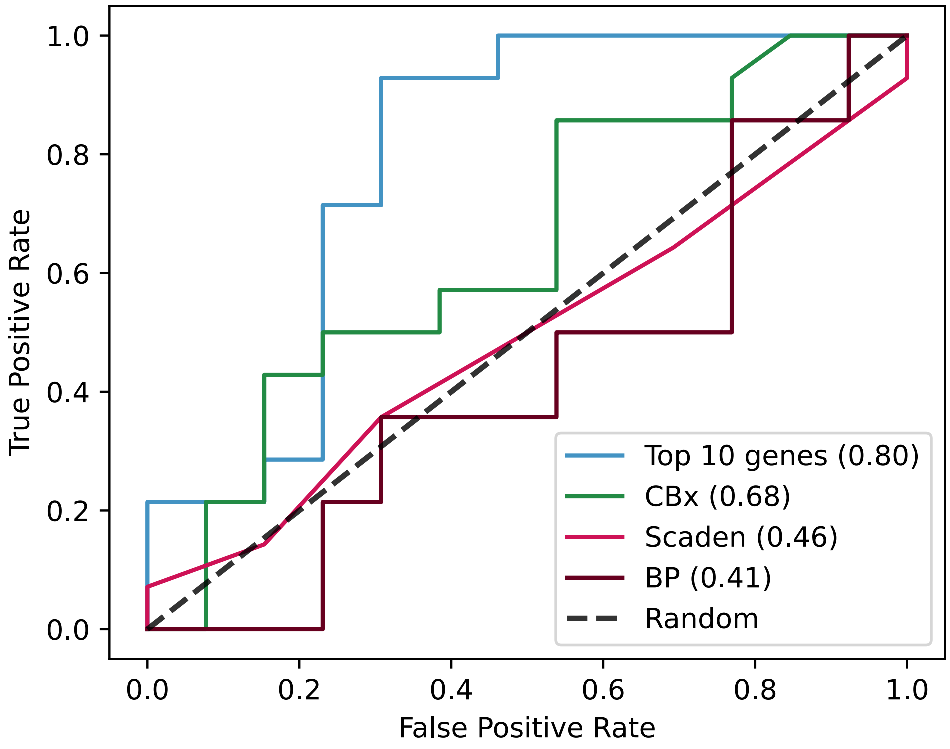

B

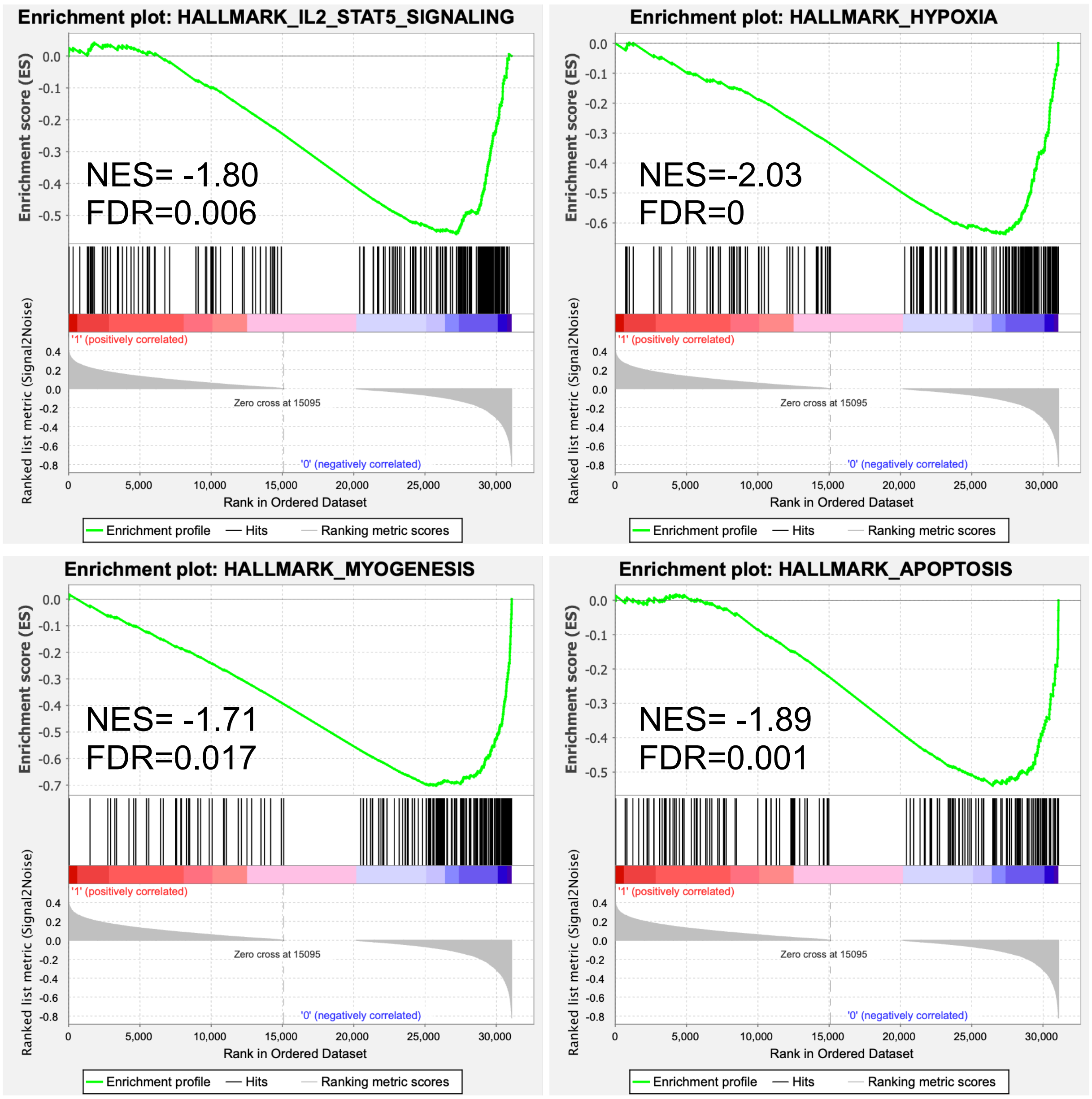

Fig. S5. (A) ROC curves for predicting anti-PD-1 response in metastatic melanoma using different feature sets. Models were trained with identical preprocessing, mutual-information feature selection, and XGBoost classification under stratified 5-fold cross-validation. Curves show ROC for CIBERSORTx (CBx; AUROC 0.68), Scaden (0.46), BayesPrism (BP; 0.41), and top 10 gene-expression features (0.80). The dashed line denotes random guessing. **(B)** GSEA analysis of the significantly down-regulated genes ($p < 0.05$) related to Fig. 6.

Fig. S6

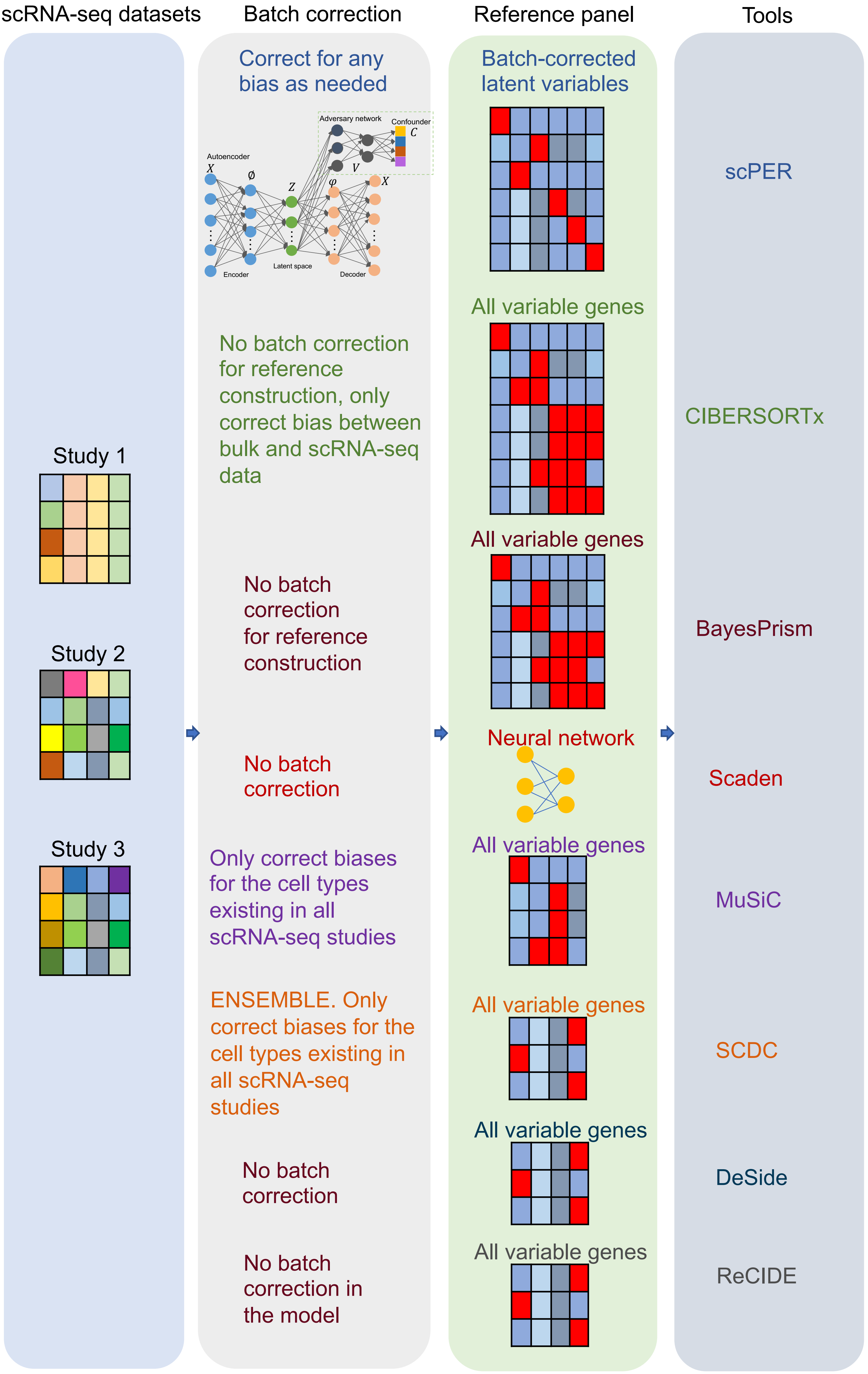

Fig. S6. Schematic of the differences between scPER and other tools.

Fig. S7

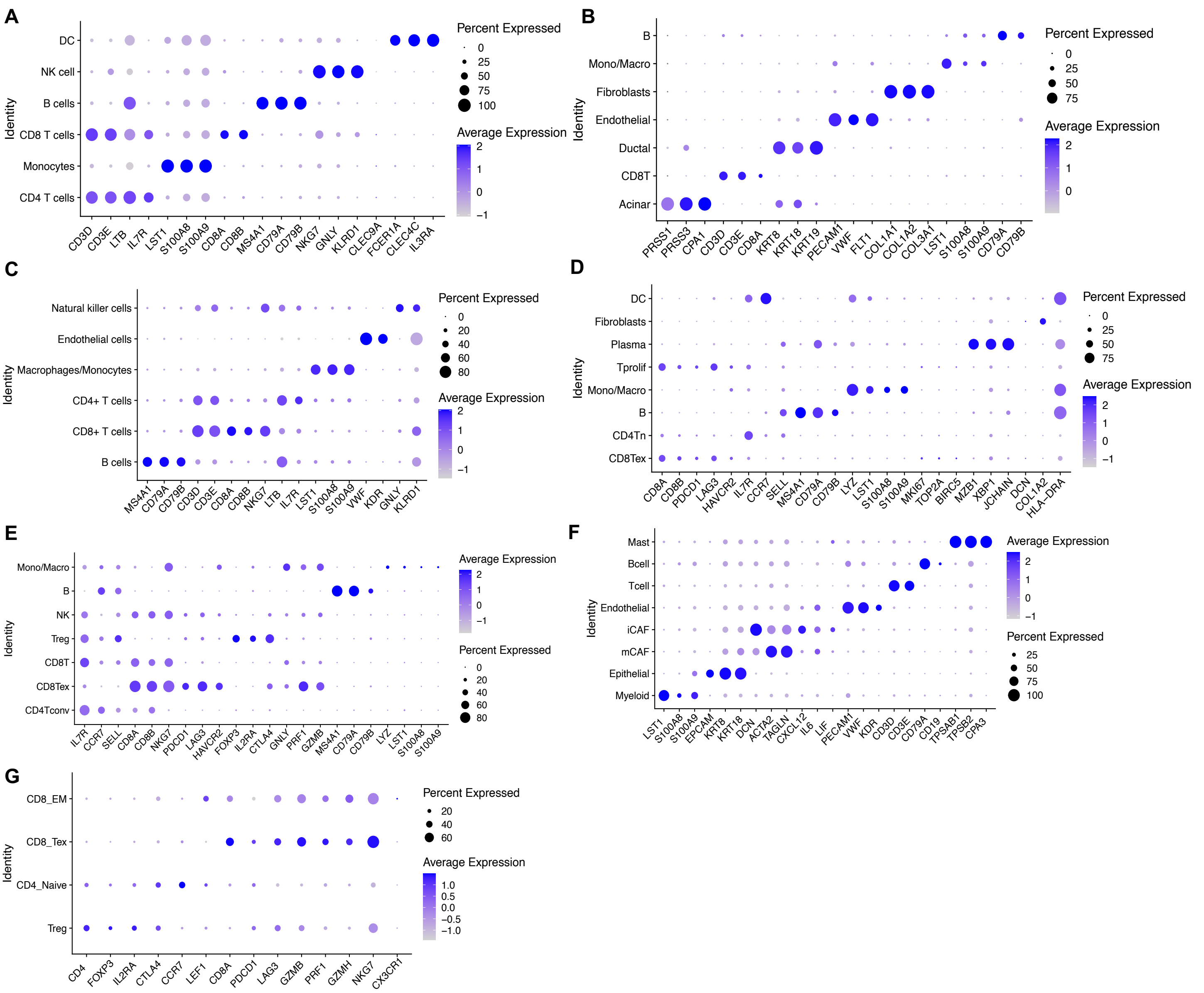

Fig. S7. Canonical marker expression used for cell-type annotation. Dot plots show average expression (color) and percent expressing cells (dot size) for representative markers across clusters in (A) PBMC (Fig. 2A), (B) pancreatic cancer (Fig. 3A), (C) cross-tissue (Fig.4A), (D-E) melanoma (Fig. 5A-B) and (F-G) bladder cancer datasets of main cell types (Fig. 6A; left panel) and T cell subtypes (Fig. 6A; right panel)

Fig. S8

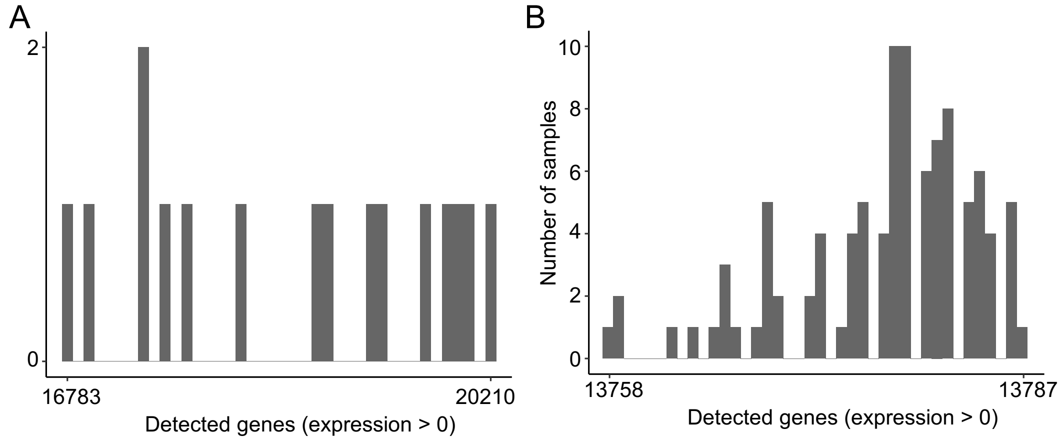

Fig S8. Gene coverage in simulated bulk RNA-seq samples constructed from scRNA-seq. Each bar represents one simulated bulk sample. Bar height denotes the number of detected genes (non-zero expression value) after aggregation and standard preprocessing. (A) Random-proportion simulation for pancreatic cancer (n = 100 samples) shows broad coverage with ~13,758–13,787 detected genes per sample. (B) Proportion-driven simulation using patient-specific cell-type proportions from the cross-tissue scRNA-seq cohort (n = 16 samples) yields ~16,783–20,210 detected genes per sample. Both strategies recover bulk-like gene coverage despite sparsity at the single-cell level, supporting the use of scRNA-seq-derived simulations for deconvolution benchmarking.

Fig. S9

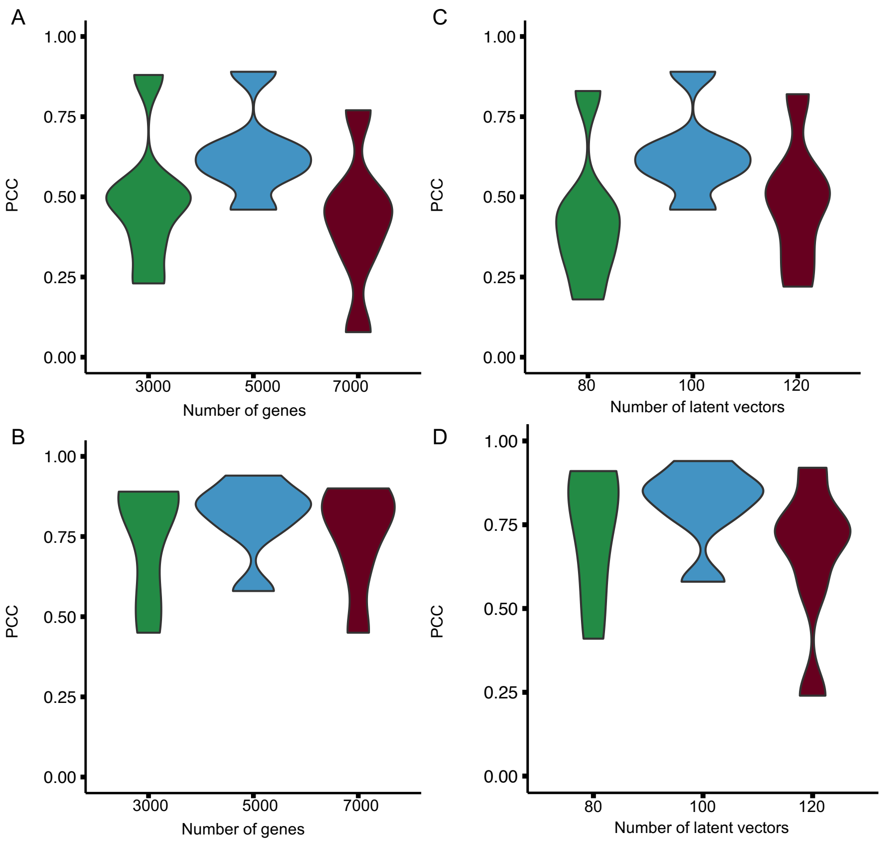

Fig. S9. Parameter sensitivity of scPER deconvolution. Violin plots show the distribution of deconvolution accuracy, evaluated by PCC between true and predicted cell-type fractions, under different feature and latent-size choices. Number of HVGs used for representation learning (3,000, 5,000, 7,000) and dimensionality of the adversarial autoencoder (80, 100, 120) for PBMC (**A&C**) and cross-tissues (**B&D**) benchmarks corresponding to the datasets used in Fig.2 and Fig. 4. All runs use identical preprocessing, training budgets, and evaluation protocols.

Table S1. Cell type proportions of bulk PBMC samples measured by flow cytometry in the original study.

Table S2. Macro-cell-type PCC, MSE and mAD to quantify the numerical closeness to ground truth across the three benchmarks.

Table S3. Mutual information scores of each cell type proportion as a feature for model prediction.

Table S4. Marker genes of all cell types in Fig. 5A

Table S5. Marker genes of all cell types in Fig. 5B
